# A Modified Fractional Maxwell Numerical Model for Constitutive Equation of Mn-Cu Damping Alloy

**DOI:** 10.3390/ma13092020

**Published:** 2020-04-26

**Authors:** Baoquan Mao, Rui Zhu, Zhiqian Wang, Yuying Yang, Xiaoping Han, Qijin Zhao

**Affiliations:** Department of Weapons and Control Engineering, Army Academy of Armored Forces, Beijing 100072, China; mbq_1965@163.com (B.M.); wangzhiqian_1990@163.com (Z.W.); lingyuxuan2012@163.com (Y.Y.); 15811060576@163.com (X.H.); zqj563954008@163.com (Q.Z.)

**Keywords:** damping alloy, constructive, fractional Maxwell

## Abstract

To better describe its constitutive relation, we need a new constitutive equation for an important nonlinear elastic material, Mn-Cu damping alloy. In this work, we studied the nonlinear and hysteretic characteristics of the stress-strain curve of the M2052 alloy with the uniaxial cyclic tensile test with constant strain rate. The strain rate and amplitude correlations of M2052 resembled those of nonlinear viscoelastic material. Therefore, we created a new constitutive equation for the M2052 damping alloy by modifying the fractional Maxwell model, and we used the genetic algorithm to carry out numerical fitting with MATLAB. By comparing with the experimental data, we confirmed that the new constitutive equation could accurately depict the nonlinear constitutive relation and hysteretic property of the damping alloy. Taken together, this new constitutive equation for Mn-Cu damping alloy based on the fractional Maxwell model can serve as an effective tool for further studies of the constitutive relation of the Mn-Cu damping alloys.

## 1. Introduction

Mn-Cu damping alloy is a typical twin-damping alloy. In the 1940s, Zener [1] first developed Mn-20Cu alloy. The Sonoston alloy (Mn–36.2Cu–3.49Al–3.04Fe–1.17Ni wt. %) was developed in the UK for submarine propellers, which effectively reduced the noise of the propeller [2]. International Copper Research Association (INCRA) developed the Incramute alloy (Mn–48.1Cu–1.55Al–0.27Si wt. %), which is mainly used in machines and pedestals [3]. Russia also developed the ABPOPA alloy (Mn-Cu-Al-Fe-Ni-Zn) in 1975 and achieved a good performance in the field of precision machinery manufacturing and ship equipment [4]. However, the machinability and applicable temperature range of the above Mn-Cu alloys were relatively low, until the M2052 (Mn-20Cu-5Ni-2Fe) alloy was developed by Kawahara et al. [5], who added Ni, Fe, Co, Ti and other elements to Mn-20Cu alloy. M2052 alloy has strong stiffness and machinability. Especially, it has high damping at room temperature, which broadens its application range. 

At present, M2052 alloy has been widely used in many industries. Xin [6] preliminarily explored the application of M2052 damping alloy on cradle of remote control weapon station. Wang [7] applied M2052 damping alloy to the motor support and fan housing. Baochang Liu et al. [8] added the powder of Mn-Cu damping alloy into the diamond drill, and found that the 40% Mn-Cu damping alloy drill had the same performance as the original one, but the vibration performance was reduce by 4.3%, and the drilling process was more stable. Komatsu et al. [9] described how apply the M2052 alloy as structural material in spacecraft vibration reduction. Yan, Shan et al. [10] mainly analyzed various characteristics of Mn-Cu damping alloy, and designed a turbine containing this alloy, which was proved to be effective in vibration reduction through experiments.

All real materials will dissipate a certain degree of energy in the process of cyclic deformation. This dissipation degree of some materials are small, such as steel, so it can be considered as a linear elastomer, and the generalized Hooke’s law can be used to describe its constitutive relationship. However, for some strict high-damping alloys, their dissipation degree of energy is much more, also with an elliptic hysteretic period and a more complex nonlinear damping. The nonlinear damping includes some viscoelastic damping, static hysteresis damping, and anelastic damping. Therefore, the high-damping alloys need to be described by a special constitutive model.

Hongzhao Liu et al. [11] regarded the aluminum-based damping alloy as a general viscoelastic material. Based on the measured data of energy storage modulus and loss factor of the damping alloy in the frequency domain, they developed a three-parameter constitutive relation model of the damping alloy. Guoqing Wang et al. [12] used the parabolic curve function to fit the loss factor and strain change rule of Zn-based damping alloy, and derived the nonlinear damping constitutive relation of Zn-based damping alloy from the equivalent viscosity theory. Sun [13] treated the constitutive relation of Fe-Mn damping alloy as a viscoelastic material, extended the constitutive model of linear viscoelastic damping to the three-dimensional by using tensor theory, and obtained the constitutive relation model of Fe-Mn damping alloy in the form of time increment. Haghdoust [14] et al. used the improved Masing criterion to simulate the nonlinear damping behavior of martensitic shape memory alloy, and realized the simulation verification in the user subroutine of the finite element program Abaqus. Since the existing model of Shape Memory Alloy (SMA) do not consider the internal circulation phenomenon of the stress-strain curve, Zbiciak et al. [15] proposed an explicit constitutive equation to represent the martensitic transformation by using the two-phase plastic body based on the rheological model of Grzes and Zbiciak [16]. The hysteretic curve has proved effective by ABAQUS.

There are no reports about the constitutive relationship of Mn-Cu damping alloys in previous papers. Most of them are about other damping alloys or SMA shape memory alloys. Although Mn-Cu damping alloy and SMA are twin damping alloys, but the first one do not have the shape memory effect and superelastic effect of the second one, so their constitutive relations are different.

However, it can be seen from literature [11,12,13] that the constitutive relationship of damping alloy can be well described by treating the damping alloy as a viscoelastic material. In other words, the damping alloy can regard as a viscous solid or a fluid with high stiffness. Maxwell model is one of the simplest model to describe the linear viscoelastic [17,18]. With the concept of fractional order derivative being developed, the fractional Maxwell model improved on its shortage of the nonlinear viscoelastic behavior description. The fractional Maxwell model can describe the nonlinear damping properties of materials within a wide frequency. It has been proven that it can better simulate the nonlinear viscoelastic material properties [19,20]. Zhao [21] established the fractional Maxwell fluid model of pipeline flow, studied the heat transfer of viscoelastic fluid in rectangular pipeline, and obtained the analytical solution of the model by using the method of separating variables. Hayat et al. [22] used Fourier transform method to solve the fraction Maxwell model of viscoelastic fluid under the condition of periodic oscillating plate. Zhiqian Wang et al. [23] proposed a fractional Maxwell model with quasi-state characteristic parameters to study the starting flow of viscoelastic colloids in the damping buffer. Jaishankar [24] constructed a fractional Maxwell model of shear deformation with quasi-state characteristics. By comparing with experimental data, it shows that the long-term power-law response predicted by the model is in good agreement with experimental data within a certain time scale. Stankiewicz [25] built a fractional Maxwell model (FMM), and deduced the expression of relaxation modulus. The curve of experiment relaxation modulus was used to determine the coefficients of FMM by piecewise curve-fitting method. Through the relaxation test of carrot root, the good fitting effect of FMM to real biological material was verified.

In order to study the nonlinear constitutive relation of Mn-Cu damping alloy, this paper takes M2052 alloy as the analysis object, through the uniaxial cyclic tensile test with constant strain rate to analysis the nonlinear and hysteresis of its stress strain curve. Then this study based on the fractional Maxwell model deduced a modified fractional Maxwell model for M2052 damping alloy, and combined with the damping mechanism of Mn-Cu damping alloy to build the governing equation. At last, we simulated the nonlinear constitutive relationship of damping alloy and compared with experimental curves.

## 2. Uniaxial Cyclic Tensile Test with Constant Strain Rate

Firstly, in order to study the nonlinear constitutive relationship of Mn-Cu damping alloy, the uniaxial cyclic tensile test at constant strain rate was be used on the damping alloy by MTS universal testing machine. Then the hysteresis stress-strain curve of the material at different strain rates and strain amplitude values can be obtained, and will provide data for the model simulation and verification.

### 2.1. Test Materials and Equipment

The composition of M2052 alloy used in the test is Mn-22.1Cu-5.24Ni-1.93Fe (mass%), and the main performance indexes are shown in the following Table 1.

**Figure 1:**
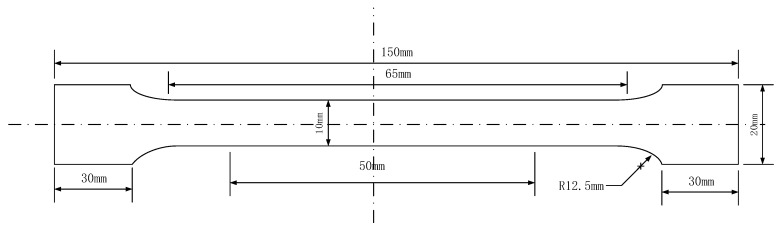
Schematic diagram of uniaxial tensile sample.

On M2052 steel plate after heat treatment, the required 150 mm × 20 mm × 2 mm tensile samples were cut by wire electrical discharge machining, and the detailed size are shown in Figure 1.

**Figure 2:**
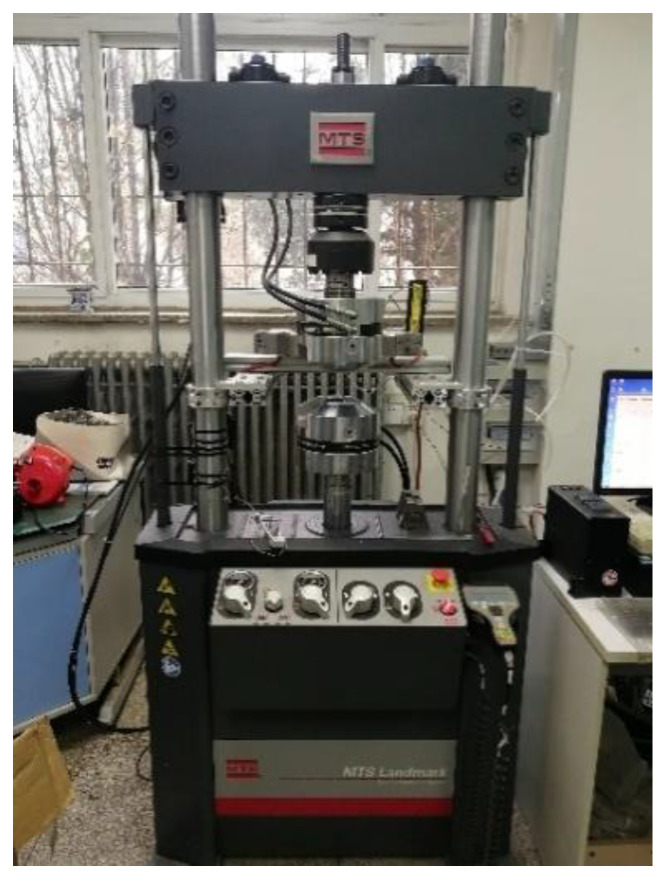
MTS-Landmark 810 universal testing machine.

**Figure 3:**
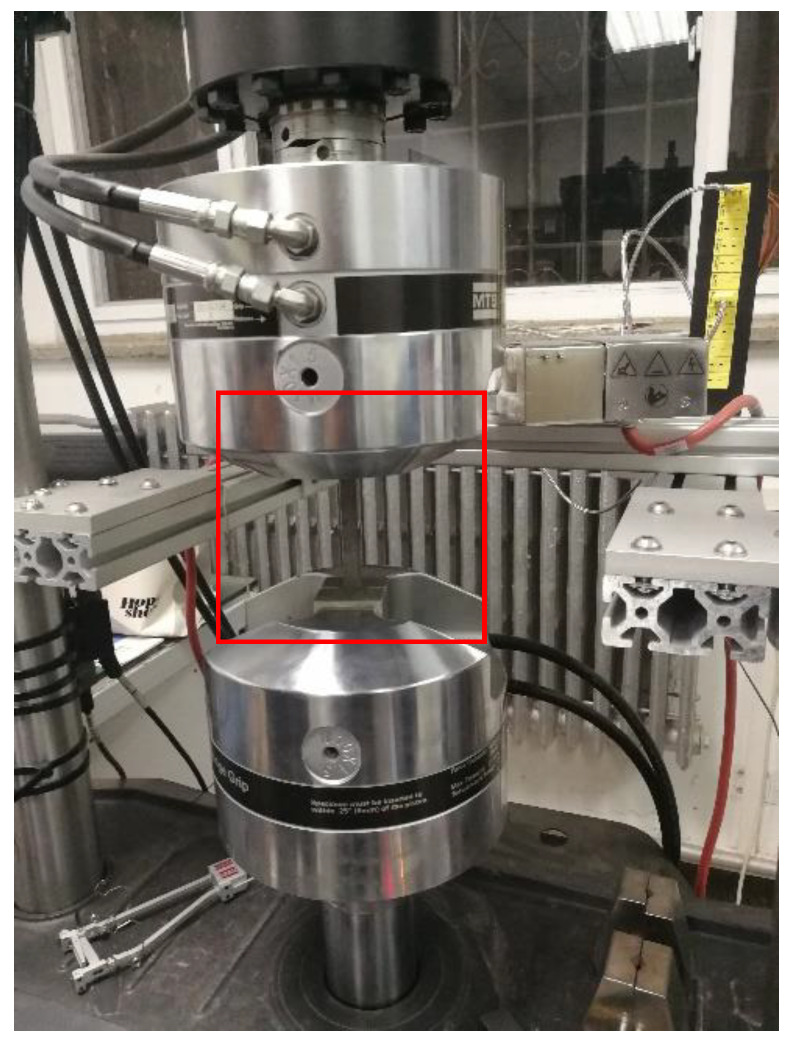
Specimen installation diagram.

The test equipment choose the MTS-Landmark 810 universal testing machine produced by USA MTS Company (Minneapolis, MN, USA), as shown in Figure 2. The bearing capacity range is 0–100 kN, the test sample frequency is 10 Hz, the length of the extensometer is 50 mm, and the installation details are shown in Figure 3.

### 2.2. Experiment Scheme and Result Analysis

Due to the high strength and damping capacity of M2052 alloy, it is often used in industry as anti-vibration structural parts. It should not produce plastic deformation, so the cyclic strain amplitude of test should not exceed 0.2%. Three types of uniaxial cyclic tensile tests were carried out. Type 1: A constant strain rate of 0.0025%/s was performed on the first tensile sample, with three different cycle strain amplitudes of 0.05%, 0.1% and 0.15%. Type 2: A constant strain rate of 0.005%/s was performed on the second tensile sample, with three different cycle strain amplitudes of 0.05%, 0.1% and 0.15%. Type 3: A constant strain rate of 0.01%/s was performed on the third tensile sample, with three different cycle strain amplitudes of 0.05%, 0.1% and 0.15%. All tests’ initial preload was 0.8 kN, and the loading and unloading process was controlled by computer program, at last outputting the stress-strain curve.

**Figure 4:**
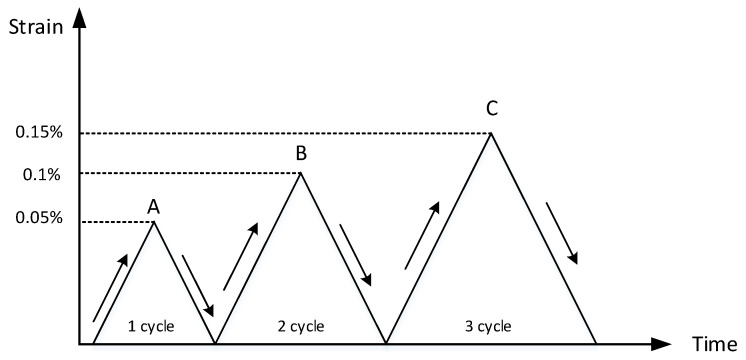
The schematic diagram of test loading-unloading process.

The tests were carried out only under strain control. In each type test, the strain rate was fixed. Firstly, stretching the sample to 0.05% strain amplitude, and then unloading it. After returning to the initial value, stretching it to 0.1% strain amplitude, and then unloading it. Finally, stretching the sample to 0.15% strain amplitude and then unloading it. The schematic diagram of test loading-unloading process is in Figure 4.

**Figure 5:**
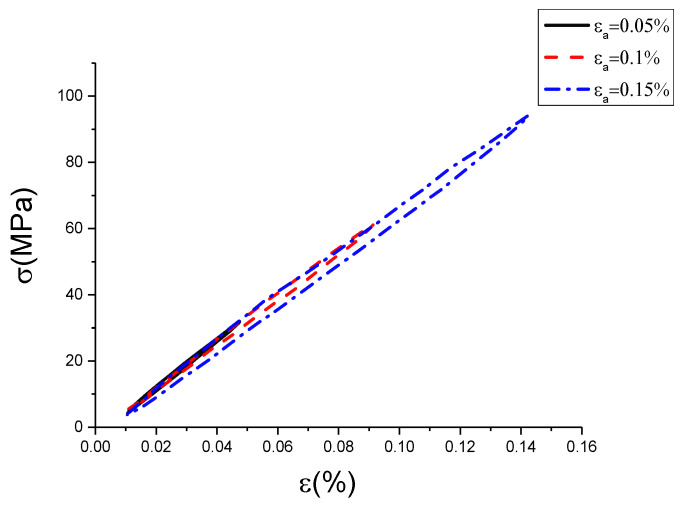
Hysteresis curves with different strain amplitudes at strain rate of 0.0025%/s (σ is stress, ε is strain, $\varepsilon_{a}$ is strain amplitude).

**Figure 6:**
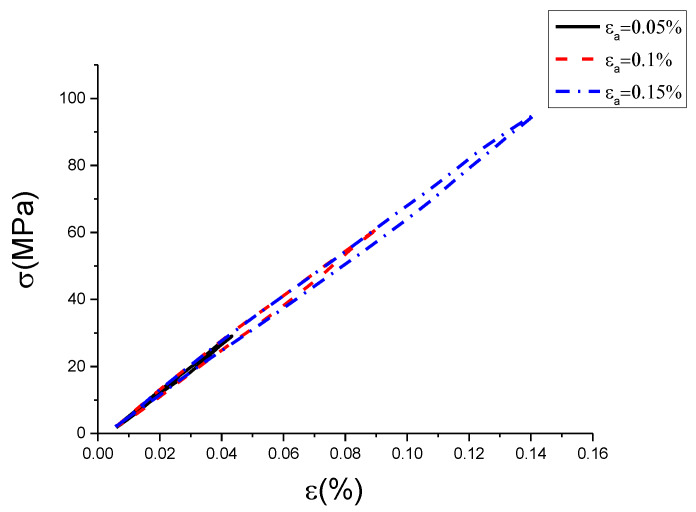
Hysteresis curves with different strain amplitudes at strain rate of 0.005%/s (σ is stress, ε is strain, $\varepsilon_{a}$ is strain amplitude).

**Figure 7:**
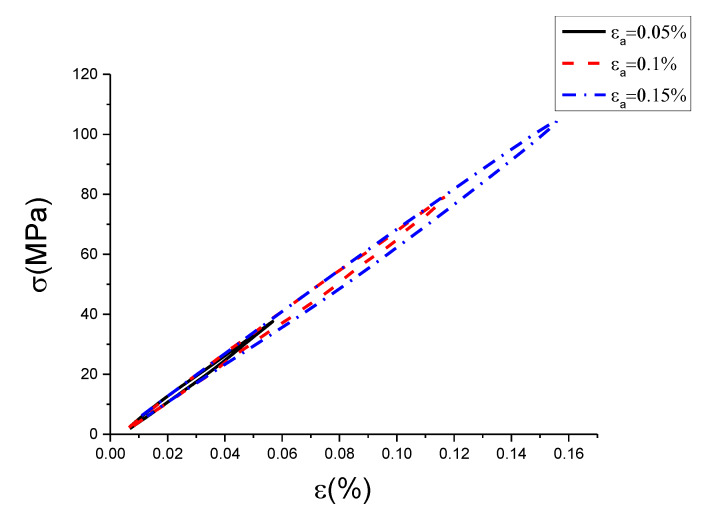
Hysteresis curves with different strain amplitudes at strain rate of 0.01%/s (σ is stress, ε is strain, $\varepsilon_{a}$ is strain amplitude).

The raw data contained some noise, which led hardly to show the characteristics of hysteresis curves with different conditions. Therefore, this work used the wavelet filtering method to filter and smooth the experimental data of uniaxial cyclic tensile tests. The result is shown in Figure 5, Figure 6 and Figure 7. In addition, the results of the original test data were put into the supplementary material (Figures S1–S3 and Tables S1 and S2).

Figure 5, Figure 6 and Figure 7 are hysteresis curves with the same strain rate but different strain amplitude. It can be seen from Table 2 and Table 3 that at the same strain rate, the hysteretic area increased with strain amplitude, and the slope of the curve decreased with strain amplitude. The hysteretic area represents the damping performance of the damping alloy. This indicates that the damping capacity is positively correlated with the amplitude within the elastic range, which has been proved by the literature [26]. However, it is found from Figure 5, Figure 6 and Figure 7 that the strain amplitude of hysteresis curve was generally not equal to the set strain value, which is due to the error caused by the response gain of the hydraulic servo valve, but it would not affect trend analysis. The strain amplitude error between the set value and the measured value of each cycle is listed as shown in Table 4.

From Table 4, we can see the measured value of strain amplitude was not equal to the set value. The error value between the measured and the set value was not the same, but all little. The relative error ranged between 0.039% and 0.175%. The measured strain amplitude at the strain rate of 0.0025%/s and 0.005%/s were less than the set value, and it was greater than the set value at the strain rate of 0.01%/s. Because the error caused by response gain of hydraulic servo valve was small, the measured strain amplitude at the strain rate of 0.0025%/s and 0.005%/s were also close. However, for the measured strain amplitude at the strain rate of 0.01%/s, the error caused by response gain of the hydraulic servo valve was large. Although the measured value of strain amplitude is inconsistent with the set value, the measured strain amplitude at each cycle with the same strain rate was still monotonically increasing. It can be concluded from Table 2 and Table 3 that with the increase of strain amplitude, the hysteresis area increased correspondingly and the slope decreased correspondingly. Therefore, the inconsistency between measured and set value will not influence the trend analysis of hysteresis curve versus strain amplitude.

Since the strain amplitudes of all tests are different, it is usually impossible to analyse the effect of the strain rate change on the hysteretic curve. However, the measured strain amplitudes at the strain rates of 0.0025%/s and 0.005%/s are close to each other, so their measured strain amplitudes can be approximately equal. It can be seen from the data in Table 2 and Table 3 that the strain rate has a certain influence on the hysteretic curve, but the specific law still needs further experimental study.

Because the error between the measured strain amplitude and the set value can be neglected, the original set value of strain amplitude is still used to describe the problem easily. Moreover, the use of measured data does not affect the fitting of the numerical model, but can avoid the loss of useful data points due to operations such as filtering and smoothing. Therefore, this paper only uses the measured data after smoothing to analyze the results of test, and the measured data without filtering is still used in the subsequent research.

## 3. Fractional Maxwell Model

### 3.1. Establishment of Fractional Maxwell Model

The basic component of the fractional-order viscoelastic model is the spring-pot element, which is a fractional-order model between the spring that represent pure elasticity and the Newton’s viscoelastic model that represent pure viscosity. Its constitutive relation can be written as [20]:

(1) $$\sigma =\kappa D_{t}^{\alpha }\varepsilon$$

where, κ is the quasi-property [27], whose unit is $Pa\cdot s^{\alpha }$, and its expression is Equation (2) [28].

(2) $$\kappa =E\left(\frac{\eta^{\alpha }}{E^{\alpha }}\right)$$

This $\tau =\frac{\eta^{}}{E^{}}$ is called relaxation time, η is the viscosity and E is the spring stiffness [29].

**Figure 8:**
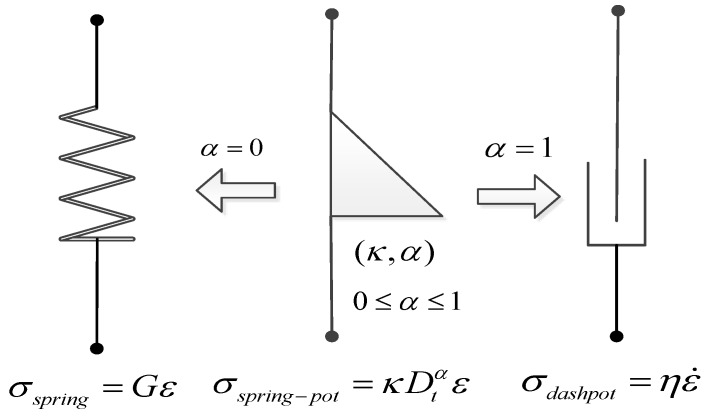
Schematic diagram of spring-pot element.

Quasi-property is the numerical measurement of a dynamic process, that it is not a simple material property. It relates with the elastic modulus, stress, time, viscosity and other physical quantities, and also associated with mathematical quantities such as fractional index (α). As shown in Figure 8, if α=0, the spring-pot element can be simplified as a spring element (σ=Eε, κ=E). If α=1, the spring-pot element simplified to a dashpot element ($\sigma =\eta \dot{\varepsilon }$, κ=η). It can be seen that the quasi-property makes the model parameters more closely to the actual research object.

**Figure 9:**
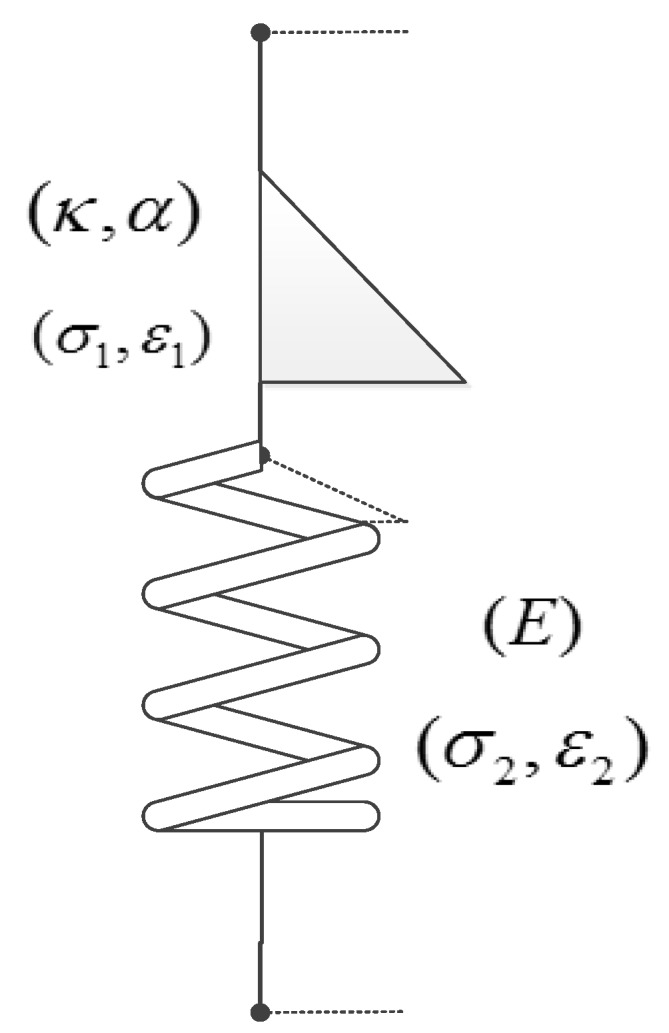
Fractional Maxwell model of M2052 damping alloy.

One spring-pot mechanism element $\left(\sigma_{1},\varepsilon_{1}\right)$ in series with one spring element $\left(\sigma_{2},\varepsilon_{2}\right)$ form the fractional Maxwell model of M2052 damping alloy (as shown in Figure 9). κ represents the quasi-property of spring-pot. α is the fractional order and 0≤α≤1. E represent the Young’s modulus of M2052 alloy. $\sigma_{1}$ is the stress of spring-pot. $\varepsilon_{1}$ is the strain of spring-pot. $\sigma_{2}$ is the stress of spring. $\varepsilon_{2}$ is the strain of spring. σ is the stress of M2052 alloy. ε is the strain of M2052 alloy. It can be obtained by Maxwell model relation:

(3) $$\sigma_{1}=\sigma_{2}=\sigma$$

(4) $$\varepsilon_{1}+\varepsilon_{2}=\varepsilon$$

Substitute Equation (1) into Equation (3) and Equation (4) to get:

(5) $$\kappa D_{t}^{\alpha }(\varepsilon -\varepsilon_{2})=E\varepsilon_{2}$$

The constitutive equation of fractional Maxwell model is:

(6) $$\sigma +\frac{\kappa }{E}D_{t}^{\alpha }\sigma =\kappa D_{t}^{\alpha }\varepsilon$$

### 3.2. The Reduces Vibration Mechanism of Mn-Cu Damping Alloy

In order to establish the governing equation of M2052 damping alloy according to the law of energy conservation, the mechanism of energy consumption of M2052 damping alloy must be understood. The high damping characteristics of Mn-Cu alloy are derived from the fact that in the process of melting cooling at high temperature, paramagnetism turns to anti-ferromagnetism after Neel point, and anti-ferromagnetic martensite twins are generated. The relaxation process of the twin boundary leads to the consumption of external energy. The parent phase of Mn-Cu damping alloy is austenite, which contains martensite twins at room temperature, so it has high damping characteristics at room temperature.

**Figure 10:**
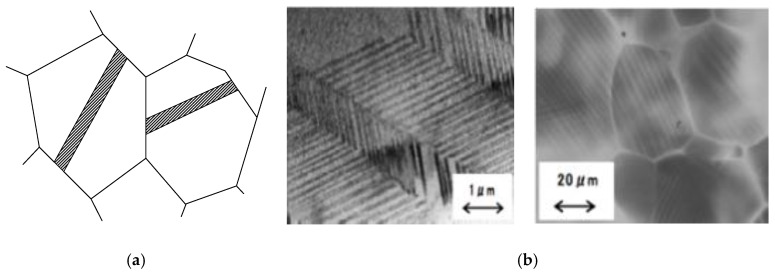
Microstructure of twins. (**a**) Schematic diagram of twin microstructure; (**b**) Electron micrograph of twin structure.

The microstructure of twin type damping alloy under external force as shown in Figure 10, from 20-micron scale under the electron microscope figure can see a horse twin throughout the austenite grain size. The 1-micron scale of damping alloy under the electron microscope can see its internal grain subdivide to many fine twins, the twins stayed parallel and cross state, when under stress.

**Figure 11:**
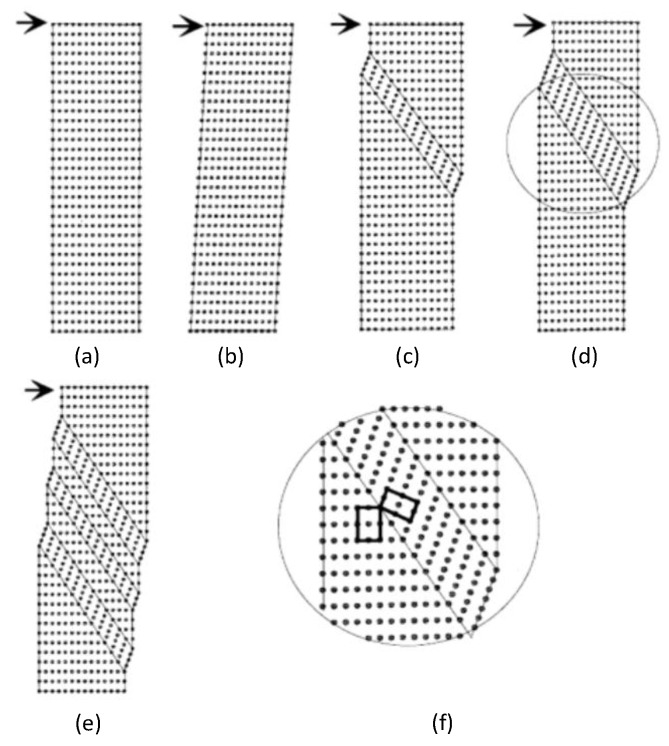
Twins movement under stress [30]. (**a**) The original lattice structure. (**b**) The deformed lattice structure. (**c**) The twin crystal lattice structure. (**d**) The larger twin crystal lattice structure. (**e**) The divided of twin crystal lattice structure. (**f**) An enlarged local view of the twin boundary.

Figure 11 is a schematic diagram of the movement of twins under stress [30]. Figure 11a is the lattice structure of the alloy without stress, and the points in the figure represent the metal atoms. When the shear stress was applied, the lattice structure began to deform, as shown in Figure 11b. When the stress reached a certain degree, twins began to generate, as shown in Figure 11c. As the stress increased, the twin band also began to increase and became a large twin crystal, as shown in Figure 11d. The deformed part and the undeformed part of the lattice formed a symmetrical relationship on the twin boundary, and the lattice direction of the twin band all changed, as shown in Figure 11f. Moreover, just because of this lattice redirection way, the energy could be absorbed. As the stress continued to increase, the large twin crystal began to differentiate into several small twin crystal, as shown in Figure 11e.

**Figure 12:**
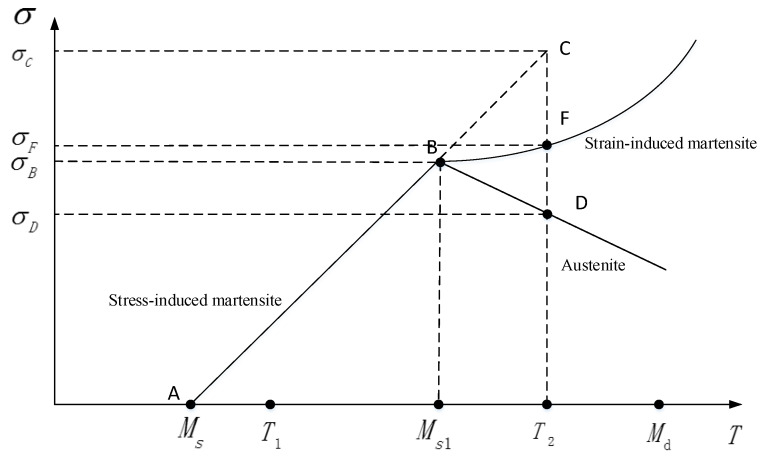
The relation of stress-induced martensite and strain-induced martensite. (σ is stress. $\sigma_{B}$ is the critical stress of stress-induced martensite. $\sigma_{C}$ is the nominal stress of stress-induced martensite at T_2_. $\sigma_{F}$ is the stress of strain-induced martensite at T_2_. $\sigma_{D}$ is the stress required for the transformation of austenite. T is temperature. Ms is the start temperature of the martensitic transformation. Md is the end temperature of the martensitic transformation. T_1_ is the room temperature.).

M2052 alloy contains martensite twins and austenite at room temperature, and the austenite is the parent phase. When the alloy is subjected to stress at room temperature, the stress-induced martensitic transformation will occur. This new martensite is called the stress-induced martensite. Since the martensitic transformation, there will be friction between the martensitic phase and austenitic phase, also between the original martensitic phase and new martensitic phase, which is also a reason for the high damping of M2052 alloy. The martensitic phase transformation includes two types: stress induced martensitic transformation and strain induced martensitic transformation. The relationship between them is shown in Figure 12.

As shown in Figure 12. When $M_{S}<T<M_{S1}$ and $0<\sigma <\sigma_{B}$, the martensitic phase transformation is mainly induced by stress (AB), and the stress and temperature show a linear relationship in the elastic range. When $M_{S1}<T<M_{d}$ and $\sigma_{B}<\sigma$, for the stress exceeding the yield strength of austenite, the plastic deformation will occur. The martensitic transformation generated by the deformation is called strain-induced martensite (BF). In addition, as the temperature increases, some martensite will begin to transform into austenite (BD). When the temperature is greater than $M_{d}$, the martensite will no longer be produced, and all phase will become austenite. Since M2052 alloy works at room temperature and plastic deformation is not allowed, stress-induced martensitic transformation will occur under the stress.

### 3.3. Governing Equation

Therefore, from the perspective of conservation of energy, under the condition of constant temperature and thermal insulation, the work done by external force all into strain energy and it should be equal to the elastic potential energy and internal energy. Moreover, the internal energy is due to the twin’s relaxation movement and the interface slip between stress-induced martensite and austenite phase [31]. At present, it is generally considered that the friction energy between martensite and austenite, martensite and martensite is small and can be ignored for the time being, so it is assumed that the strain energy is the sum of elastic potential energy and twin energy. The expression is shown in Equation (7).

(7) $$W_{S}=W_{E}+W_{T}$$

where $W_{S}$ is the strain energy per unit volume, $W_{E}$ is the elastic potential energy per unit volume, and $W_{T}$ is the twin energy per unit volume.

**Figure 13:**
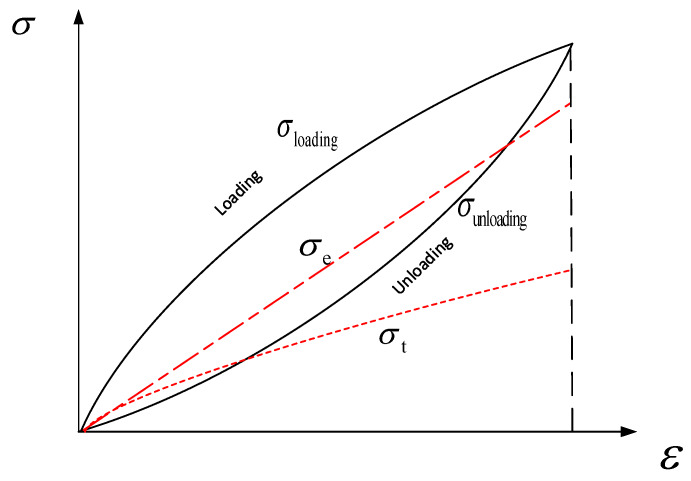
Schematic diagram of energy conversion.

The schematic diagram of energy conversion was shown in Figure 13. $\sigma_{\mathrm{loading}}$ is the stress of loading stage. $\sigma_{\mathrm{unloading}}$ is the stress of unloading stage. $\sigma_{e}$ is elastic stress. $\sigma_{t}$ is the stress of twin. Each energy density is the area, which is surround by each curve and x-axis. The area between $\sigma_{\mathrm{loading}}$ and $\sigma_{\mathrm{unloading}}$ is the loss of energy per unit volume. The strain energy per unit volume is equal to the $\sigma_{e}$ surrounding area and $\sigma_{t}$ surrounding area combined.

According to the J2 deformation theory, Hooke’s law and Equations (1)–(4), we can obtain blow equations [32] under the unit volume:

(8) $$W_{S}=\int_{0}^{t}\sigma \dot{\varepsilon }dt,W_{E}=\frac{1}{2}E{\left(\varepsilon_{2}\right)}^{2},W_{T}=\int_{0}^{t}\sigma_{1}{\dot{\varepsilon }}_{1}dt.$$

Then taking Equation (8) into Equation (7) we can receive Equation (9).

(9) $$\int_{0}^{t}\sigma \dot{\varepsilon }dt=\frac{1}{2}E{\left(\varepsilon -\frac{1}{k}D_{t}^{-\alpha }\sigma \right)}^{2}+\frac{1}{k}\int_{0}^{t}\sigma D_{t}^{1-\alpha }\sigma dt$$

The formula can convert into Equation (10):

(10) $$\int_{0}^{t}\sigma \dot{\varepsilon }dt=\frac{1}{2}\frac{\sigma^{2}}{E}+\frac{1}{k}\int_{0}^{t}\sigma D_{t}^{1-\alpha }\sigma dt$$

The above equation is the governing equation of fractional Maxwell model of M2052 damping alloy.

In the governing equation, when t≤0, then σ(t)=0,ε(t)=0 ; when t>0, σ(t) and ε(t) is monotonically increasing in the loading segment and monotonically decreasing in the unloading segment. Therefore, the initial value and boundary conditions of the governing equation are shown in Equation (11):

(11) $$\{\begin{array}{c} \sigma (0)=0,t>0\\ \varepsilon (0)=0,t>0\\ E=68.5GPa\\ \begin{array}{c} \sigma (t_{i+1})>\sigma (t_{i}),\dot{\varepsilon }>0\\ \sigma (t_{i+1})<\sigma (t_{i}),\dot{\varepsilon }<0 \end{array} \end{array}$$

### 3.4. Numerical Solution

Taking the derivatives of both sides of the governing Equation (10) with respect to time, we can get the below equations.

(12) $$\sigma \dot{\varepsilon }=\frac{\sigma \dot{\sigma }}{E}+\frac{1}{k}\sigma D_{t}^{1-\alpha }\sigma$$

(13) $$\dot{\varepsilon }=\frac{\dot{\sigma }}{E}+\frac{1}{k}D_{t}^{1-\alpha }\sigma$$

In order to solve the above equation, the fractional derivative is discretized by the finite difference method.

Define $t_{m}=m\Delta t$, m=0,1,2,…,K, 0≤k≤K; $\Delta t=\frac{T}{K}$ stands the time step. According to the definition of fractional calculus of Caputo [33], when 0<h<1, then a=0,n=1, $D_{t}^{h}\sigma (t)$ (0<h<1) can be written as:

(14) $$D_{t}^{h}\sigma (t_{k})=\frac{1}{\Gamma (1-h)}\sum_{i=0}^{k-1}\int_{t_{i}}^{t_{i+1}}\frac{\partial \sigma (s)}{\partial s}\frac{ds}{{\left(t_{k}-s\right)}^{h}}$$

where, according to the finite difference method, $\frac{\partial \sigma (t)}{\partial t}$ and $\int_{t_{i}}^{t_{i+1}}\frac{ds}{{\left(t_{k}-s\right)}^{h}}$ can be written in the interval $\left[t_{i},t_{i+1}\right]$ as:

(15) $$\frac{\partial \sigma (t)}{\partial t}=\frac{\sigma (t_{i+1})-\sigma (t_{i})}{\Delta t}+o(\Delta t)$$

where $\frac{\partial \sigma (t)}{\partial t}\approx \frac{\sigma (t_{i+1})-\sigma (t_{i})}{\Delta t}$ is the forward difference form of $\frac{\partial \sigma (t)}{\partial t}$; o(Δt) is the error of approximation.

(16) $$\begin{array}{ll} \int_{t_{i}}^{t_{i+1}}\frac{ds}{{\left(t_{k}-s\right)}^{h}} & =-\frac{1}{1-h}{\left(t_{k}-s\right)}^{1-h}|{}_{t_{i}}^{t_{i+1}}\\ & =-\frac{1}{1-h}\left[{\left(t_{k}-t_{i+1}\right)}^{1-h}-{\left(t_{k}-t_{i}\right)}^{1-h}\right] \end{array}$$

In addition, because of the idea of difference, $t_{k+1}=\left(k+1\right)\Delta t$, by the same token $t_{k},t_{i+1},t_{i}$, the above equation can be changed into:

(17) $$\begin{array}{ll} \int_{t_{i}}^{t_{i+1}}\frac{ds}{{\left(t_{k}-s\right)}^{h}} & =-\frac{1}{1-h}{\left(t_{k}-s\right)}^{1-h}|{}_{t_{i}}^{t_{i+1}}\\ & =-\frac{1}{1-h}\left[{\left(t_{k}-t_{i+1}\right)}^{1-h}-{\left(t_{k}-t_{i}\right)}^{1-h}\right]\\ & =\left[{\left(k-i\right)}^{1-h}-{\left(k-i-1\right)}^{1-h}\right]\frac{{\Delta t}^{1-h}}{1-h} \end{array}$$

Taking Equations (15) and (17) into Equation (14), and defining j=k−i we can obtain:

(18) $$\begin{array}{l} \frac{1}{\Gamma (1-h)}\sum_{i=0}^{k-1}\frac{\sigma (t_{i+1})-\sigma (t_{i})}{\Delta t}\int_{t_{i}}^{t_{i+1}}\frac{ds}{{\left(t_{k}-s\right)}^{h}}=\\ \frac{1}{\Gamma (1-h)}\sum_{i=0}^{k-1}\frac{\sigma (t_{i+1})-\sigma (t_{i})}{\Delta t}\left[{\left(k-i\right)}^{1-h}-{\left(k-i-1\right)}^{1-h}\right]\frac{{\Delta t}^{1-h}}{1-h}+o({\left(\Delta t\right)}^{r})\\ =\frac{{\left(\Delta t\right)}^{-h}}{\Gamma (2-h)}\sum_{j=1}^{k}h_{j}\left[\sigma (t_{k-j+1})-\sigma (t_{k-j})\right]+o({\left(\Delta t\right)}^{r}) \end{array}$$

The coefficient in the higher-order term r=2−h, define $h_{j}=j^{1-h}-{\left(j-1\right)}^{1-h}$, j=1,…,k.

$o({\left(\Delta t\right)}^{r})$ is the higher-order error term.

So we can get the expression of $D_{t}^{h}\sigma (t_{k})$, as shown in Equation (19).

(19) $$\begin{array}{l} D_{t}^{h}\sigma (t_{k})=\frac{{\left(\Delta t\right)}^{-h}}{\Gamma (2-h)}\sum_{j=1}^{k}h_{j}\left[\sigma (t_{k-j+1})-\sigma (t_{k-j})\right]+o({\left(\Delta t\right)}^{2-h})\\ =\frac{{\left(\Delta t\right)}^{-h}}{\Gamma (2-h)}\left[h_{1}\sigma (t_{k})-h_{k}\sigma (t_{0})+\sum_{j=2}^{k}\left(h_{j}-h_{j-1})\sigma (t_{k-j+1}\right)\right]+o({\left(\Delta t\right)}^{2-h}) \end{array}$$

Since $h_{1}=1$, the above equation can be simplified to:

(20) $$\begin{array}{ll} D_{t}^{h}\sigma (t_{k}) & =\frac{{\left(\Delta t\right)}^{-h}}{\Gamma (2-h)}\left[\sigma (t_{k})-h_{k}\sigma (t_{0})+\sum_{j=2}^{k}\left(h_{j}-h_{j-1})\sigma (t_{k-j+1}\right)\right]\\ & +o({\left(\Delta t\right)}^{2-h}) \end{array}$$

When 0<α<1, the 1−α>0, define h=1−α, taking it into Equation (20).

(21) $$\begin{array}{ll} D_{t}^{1-\alpha }\sigma (t_{k}) & =\frac{{\left(\Delta t\right)}^{\alpha -1}}{\Gamma (1+\alpha )}\left[\sigma (t_{k})-h_{k}\sigma (t_{0})+\sum_{j=2}^{k}\left(h_{j}-h_{j-1})\sigma (t_{k-j+1}\right)\right]\\ & +o({\left(\Delta t\right)}^{1+\alpha }) \end{array}$$

Where $h_{j}=j^{\alpha }-{\left(j-1\right)}^{\alpha }$, j=2,…,k.

Take Equations (15) and (21) into Equation (13), and the higher-order error terms can be omitted:

(22) $$\begin{array}{l} \dot{\varepsilon }=\frac{\sigma (t_{k+1})-\sigma (t_{k})}{E\Delta t}+\frac{1}{\kappa }\frac{{\left(\Delta t\right)}^{\alpha -1}}{\Gamma (1+\alpha )}\left\{\begin{array}{l} \sigma (t_{k})-h_{k}\sigma (t_{0})+\\ \sum_{j=2}^{k}\left(h_{j}-h_{j-1})\sigma (t_{k-j+1}\right) \end{array}\right\}\\ \left(k=1:m,i=0:k-1,j=2:k,h_{j}=j^{\alpha }-{\left(j-1\right)}^{\alpha },j=2,\ldots ,k\right) \end{array}$$

## 4. Numerical Model Simulation Analysis

### 4.1. Model Parameter Analysis

Taking the fractional Maxwell numerical model and boundary conditions into MATLAB software, and then combining with the test data determined the time step Δt = 0.1 s. We selected the more regular curve which was under strain rate 0.0025%/s and strain amplitude 0.1% as the basic experiment data and σ(0)=4.5901 MPa, ε(0)=0.00903%. Then we analyzed the influence of α and κ values on the model.

α is the fractional order coefficient, and its range is 0<α<1, and the range of quasi-properties κ can be determined by E and relaxation time according to Equation (2). Although no data on the relaxation time of M2052 alloy have been found (which can be measured by future relaxation tests), we can know when α=0, the minimum value of κ is 68.5 GPa.

In order to facilitate the analysis, we define $\kappa =C\times 10^{9}$, 68.5<C<∞ and select α=0.001,0.01,0.1,0.2,0.3,0.4,0.5,0.6,0.7,0.8,0.9, C=100,500,2500,12500.

**Figure 14:**
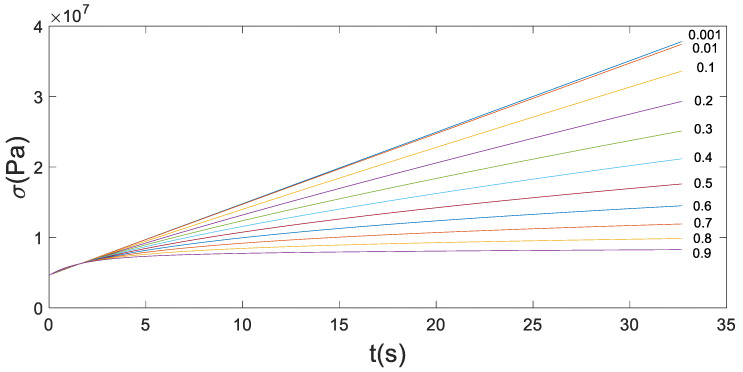
The fitting curve of stress in loading stage for fixed C = 100.

When C=100 and α=0.001,0.01,0.1,0.2,0.3,0.4,0.5,0.6,0.7,0.8,0.9, the stress-time fitting curve of the loading stage with strain rate of 0.0025%/s and strain amplitude of 0.1% is shown in Figure 14.

As can be seen from Figure 14, fractional Maxwell can simulate the nonlinear curve of the convex function in the loading section. Moreover, with the increase of the α, the curvature is higher and higher, but the maximum stress is lower. In order to observe the influence of the κ, choose α that at the largest curvature as an invariant.

**Figure 15:**
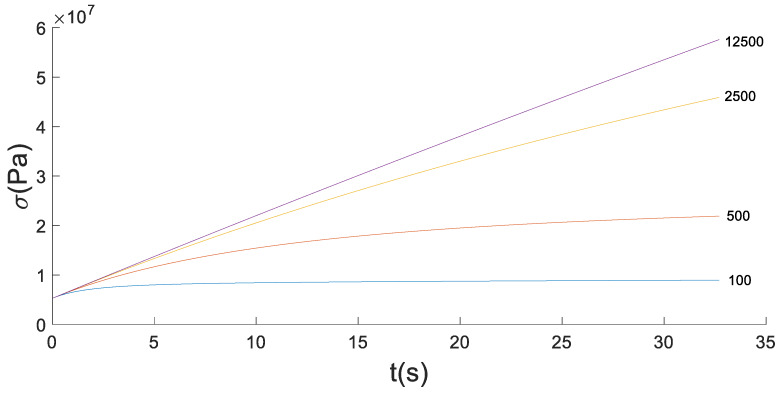
The fitting curve of stress in loading stage for fixed α=0.9.

When α=0.9 and C=100,500,2500,12500, the stress-time fitting curve of the loading stage with the strain rate of 0.0025%/s and the strain amplitude of 0.1% is shown in Figure 15.

It can be seen from Figure 15 that the increase of κ will reduce the curvature of the curve, but can significantly increase the increment of the stress over time. Therefore, there is an optimal solution between α and κ.

### 4.2. Genetic Algorithm Setting

**Figure 16:**
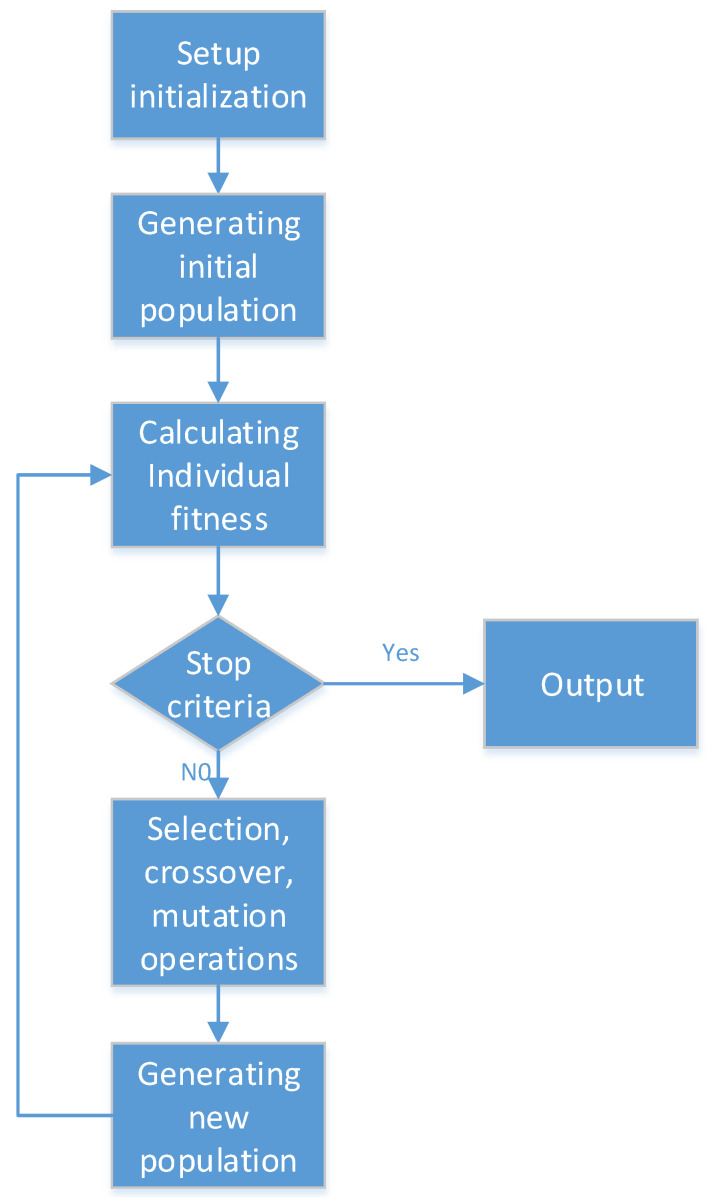
Flow chart of genetic optimization.

Since the governing Equation (21) is a difference iterative form with time as the iterative variable, the least square method or linear regression method cannot be used, so the fitting problem can be transformed into a multi-objective optimization problem. The objective function, design variables and constraint conditions are prepared by using the GA toolbox of MATLAB 2016R. The optimization process is shown in Figure 16.

Some parameters are set as follows:

(23) $$\mathrm{Objective}\;\mathrm{Function}:\;\mathrm{obj}=\frac{\overset{n}{\underset{i}{\sum }}{\left(y_{i}-{y_{i}}^{\prime }\right)}^{2}}{n}$$

$y_{i}$ stands stress value of the fitting curve corresponding to the test strain point, ${y_{i}}^{\prime }$ is the stress value of the test data, and n is the test data number.

Population size: 200, elite count: 10, crossover fraction: 0.85, the end condition is that the two optimal fitness errors of individuals are less than 1 × 10^−15^.

### 4.3. Test Data Fitting

Based on the loading section of uniaxial cyclic tensile test data and genetic algorithm, there are nine sets of data corresponding to three strain rates and three strain amplitudes can fit out the different values of α and C. Then can take them into unloading program to get the loading and unloading cycle tensile curve.

The fitting results of fractional Maxwell model are shown in Figure 17, Figure 18, Figure 19, Figure 20, Figure 21, Figure 22, Figure 23, Figure 24 and Figure 25.

**Figure 17:**
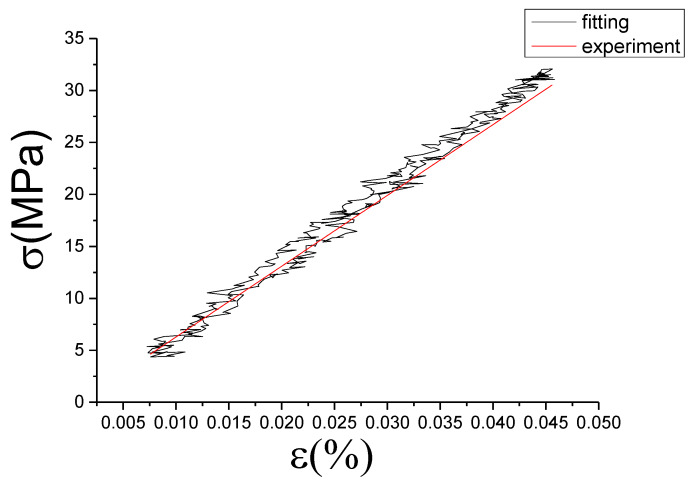
Comparison of experimental stress–strain curves and model predictions at $\dot{\varepsilon }=0.0025\%/s$ and $\varepsilon_{a}=0.05\%$. (ε(%) is strain, σ (MPa) is stress, $\dot{\varepsilon }(\%/s)$ is strain rate, $\varepsilon_{a}(\%)$ is strain amplitude, the fitting curve is red and the experiment data is black.).

**Figure 18:**
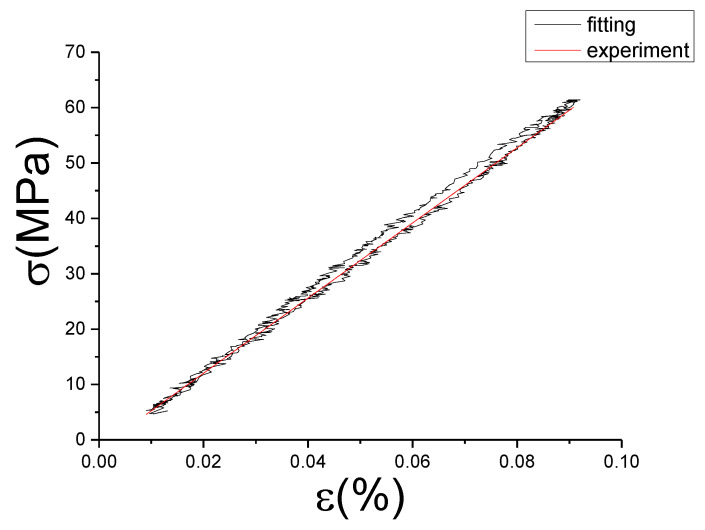
Comparison of experimental stress–strain curves and model predictions at $\dot{\varepsilon }=0.0025\%/s$ and $\varepsilon_{a}=0.1\%$. (ε(%) is strain, σ (MPa) is stress, $\dot{\varepsilon }(\%/s)$ is strain rate, $\varepsilon_{a}(\%)$ is strain amplitude, the fitting curve is red and the experiment data is black.).

**Figure 19:**
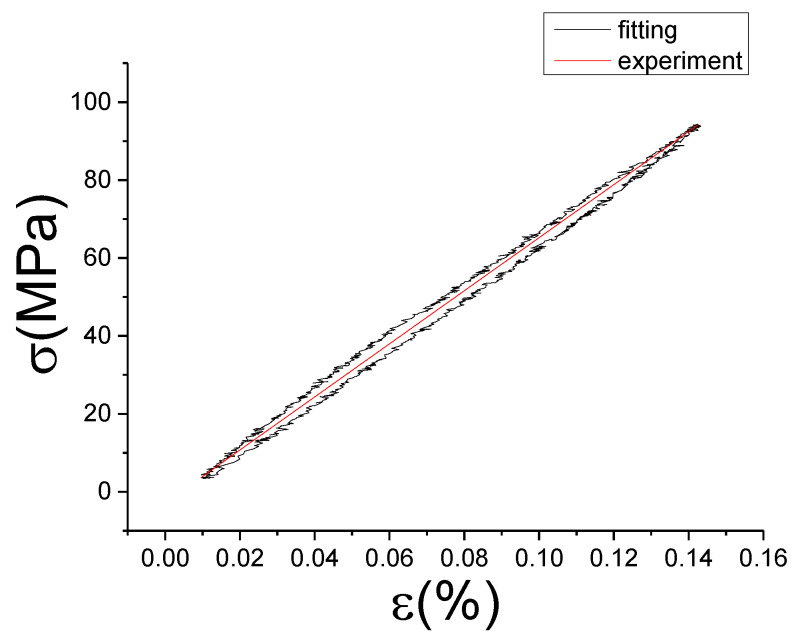
Comparison of experimental stress–strain curves and model predictions at $\dot{\varepsilon }=0.0025\%/s$ and $\varepsilon_{a}=0.15\%$. (ε(%) is strain, σ (MPa) is stress, $\dot{\varepsilon }(\%/s)$ is strain rate, $\varepsilon_{a}(\%)$ is strain amplitude, the fitting curve is red and the experiment data is black.).

**Figure 20:**
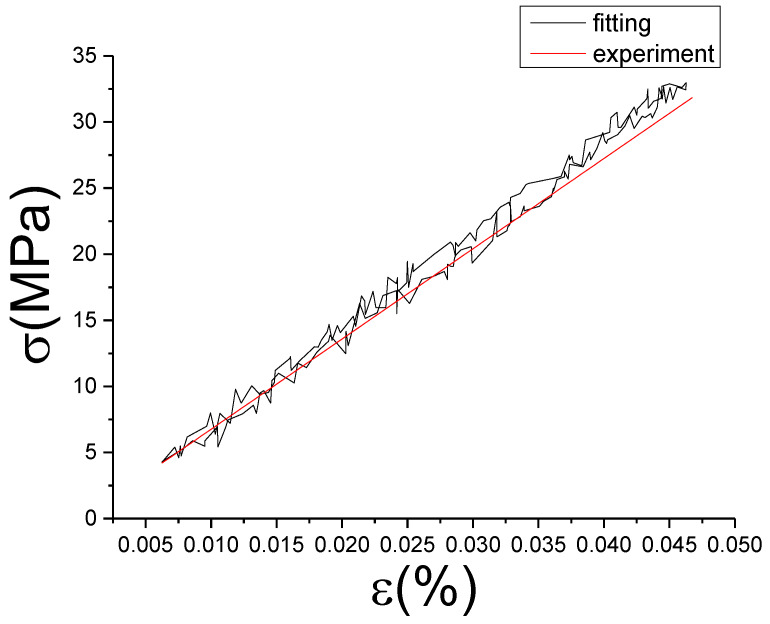
Comparison of experimental stress–strain curves and model predictions at $\dot{\varepsilon }=0.005\%/s$ and $\varepsilon_{a}=0.05\%$. (ε(%) is strain, σ (MPa) is stress, $\dot{\varepsilon }(\%/s)$ is strain rate, $\varepsilon_{a}(\%)$ is strain amplitude, the fitting curve is red and the experiment data is black.).

**Figure 21:**
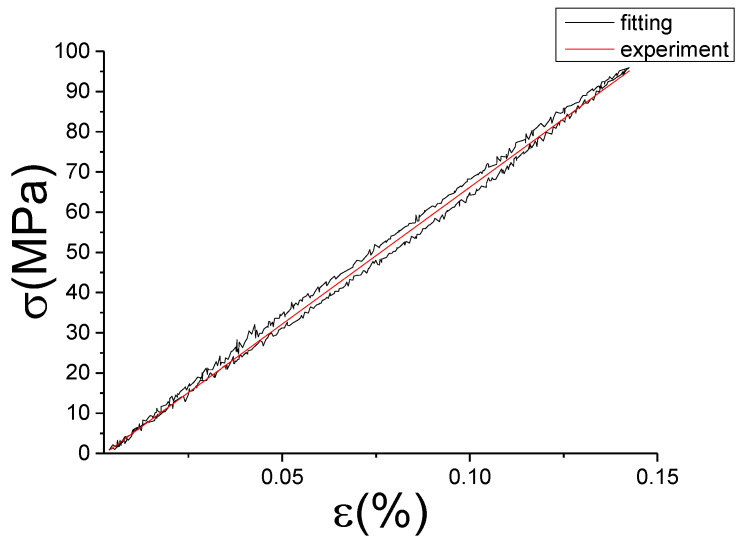
Comparison of experimental stress–strain curves and model predictions at $\dot{\varepsilon }=0.005\%/s$ and $\varepsilon_{a}=0.1\%$. (ε(%) is strain, σ (MPa) is stress, $\dot{\varepsilon }(\%/s)$ is strain rate, $\varepsilon_{a}(\%)$ is strain amplitude, the fitting curve is red and the experiment data is black.).

**Figure 22:**
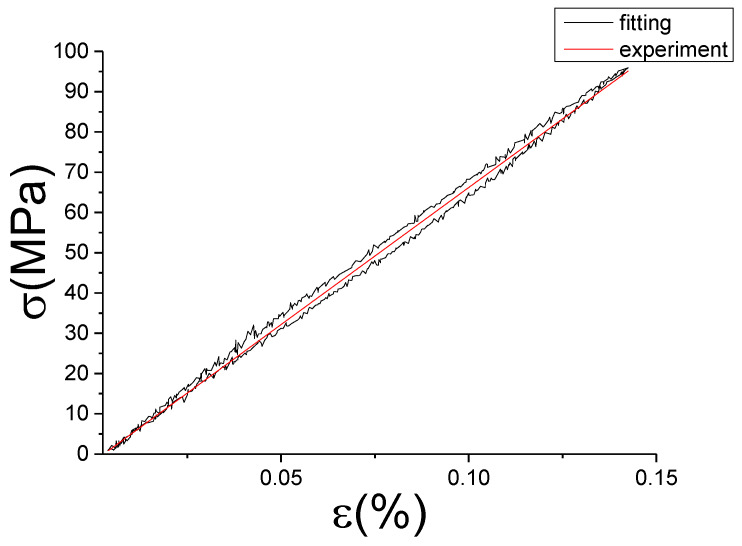
Comparison of experimental stress–strain curves and model predictions at $\dot{\varepsilon }=0.005\%/s$ and $\varepsilon_{a}=0.15\%$. (ε(%) is strain, σ (MPa) is stress, $\dot{\varepsilon }(\%/s)$ is strain rate, $\varepsilon_{a}(\%)$ is strain amplitude, the fitting curve is red and the experiment data is black.).

**Figure 23:**
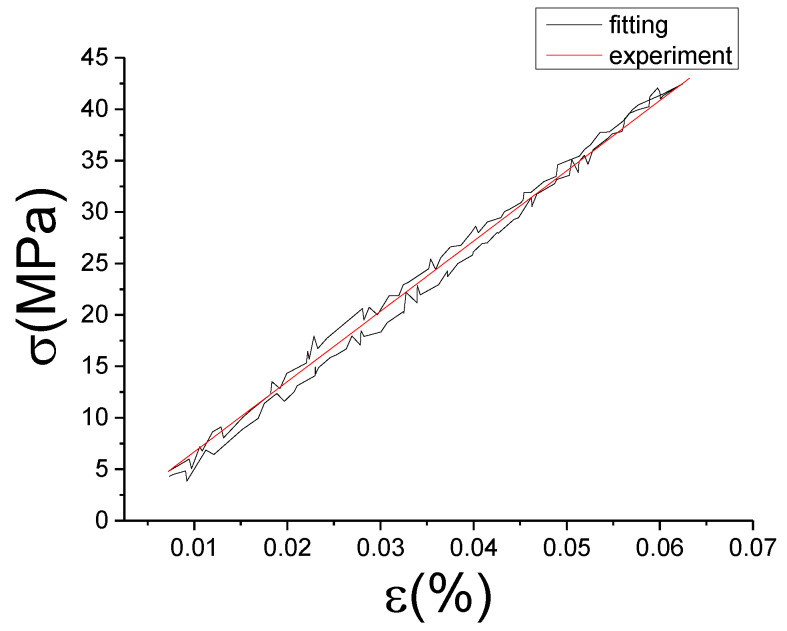
Comparison of experimental stress–strain curves and model predictions at $\dot{\varepsilon }=0.01\%/s$ and $\varepsilon_{a}=0.05\%$. (ε(%) is strain, σ (MPa) is stress, $\dot{\varepsilon }(\%/s)$ is strain rate, $\varepsilon_{a}(\%)$ is strain amplitude, the fitting curve is red and the experiment data is black.).

**Figure 24:**
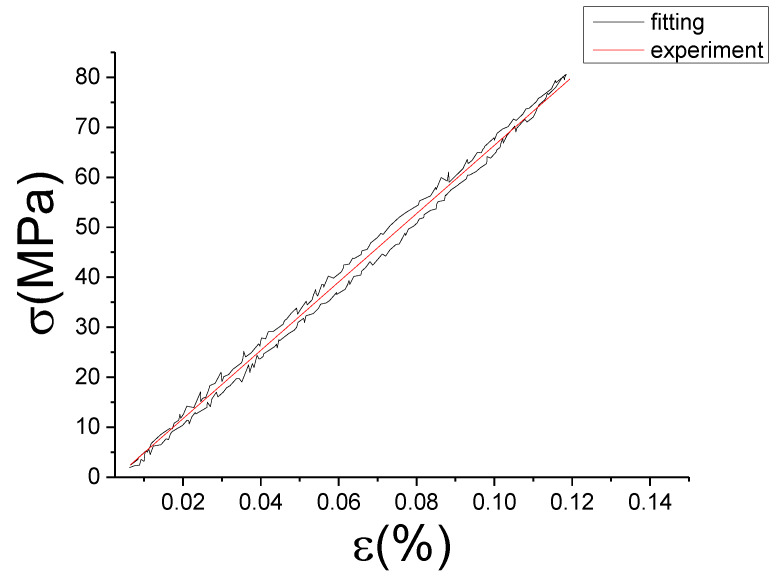
Comparison of experimental stress–strain curves and model predictions at $\dot{\varepsilon }=0.01\%/s$ and $\varepsilon_{a}=0.1\%$. (ε(%) is strain, σ (MPa) is stress, $\dot{\varepsilon }(\%/s)$ is strain rate, $\varepsilon_{a}(\%)$ is strain amplitude, the fitting curve is red and the experiment data is black.).

**Figure 25:**
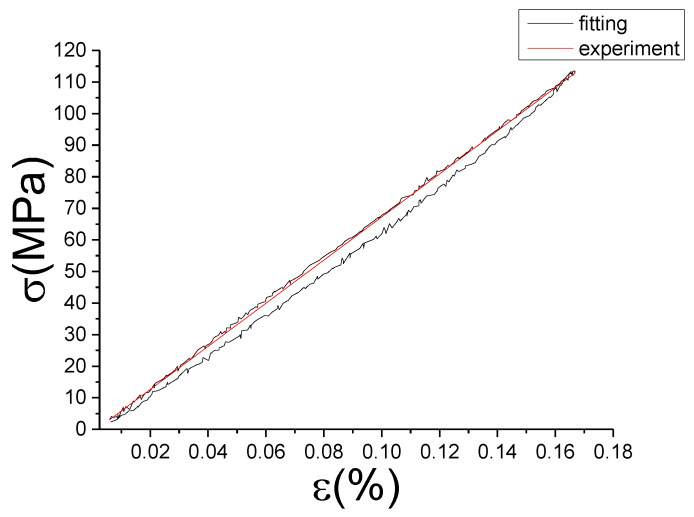
Comparison of experimental stress–strain curves and model predictions at $\dot{\varepsilon }=0.01\%/s$ and $\varepsilon_{a}=0.15\%$. (ε(%) is strain, σ (MPa) is stress, $\dot{\varepsilon }(\%/s)$ is strain rate, $\varepsilon_{a}(\%)$ is strain amplitude, the fitting curve is red and the experiment data is black.).

From Figure 17, Figure 18, Figure 19, Figure 20, Figure 21, Figure 22, Figure 23, Figure 24 and Figure 25, it can be seen that the fractional Maxwell model has a good fit for the loading section, but the unloading section has a large deviation, and the overlap of loading and unloading curves cannot reflect hysteresis loop, this is because the maximum stress value of the loading section fitting curve is smaller. Table 5 shows the fitting coefficients of fractional Maxwell model corresponding to each group of data.

It can be seen from Table 5 that each fitting iteration has a large number to ensure the credibility of the optimization and avoid falling into local optimization. The optimal fitness value (Fval) ranges from 0.79 to 3.98, which represents the approximation between the fitting curve and the test data, and the smaller Fval, the better the result gets. The values of α and C are not the same in the group 1–9. This phenomenon demonstrates that the damping capacity of M2052 is related to the strain rate and strain amplitude. At the same strain rate, with the increase of the strain amplitude, the combination α with C also makes the slope of the fitting curve larger. At the same strain amplitude, with the change of the strain rate, the combination α with C also change but no special law. The value range of α is $5.1945\times 10^{-4}—0.0125$ generally small, while C value is bigger, in the range of 7811.4–29563. Combined with Section 4.1, since the max-stress of loading stage is too big, the fractional coefficient should be decreased and the κ should be increased to achieve the minimum mean square error. This will lead to the weak nonlinear of fitting curve. It cannot conform to the characteristics of nonlinear damping alloy. Therefore, we need to modify model in Section 5. However, the fitting curve of the basic model is concentrated near the symmetry line of the test hysteresis loop, which indicates that the numerical range of the model fitting is close to the actual value. It can be used as approximate curve or equivalent curve in engineering application or when the accuracy demand is not high.

## 5. Modified Fractional Maxwell Model

### 5.1. Establishment of Correction Term

**Figure 26:**
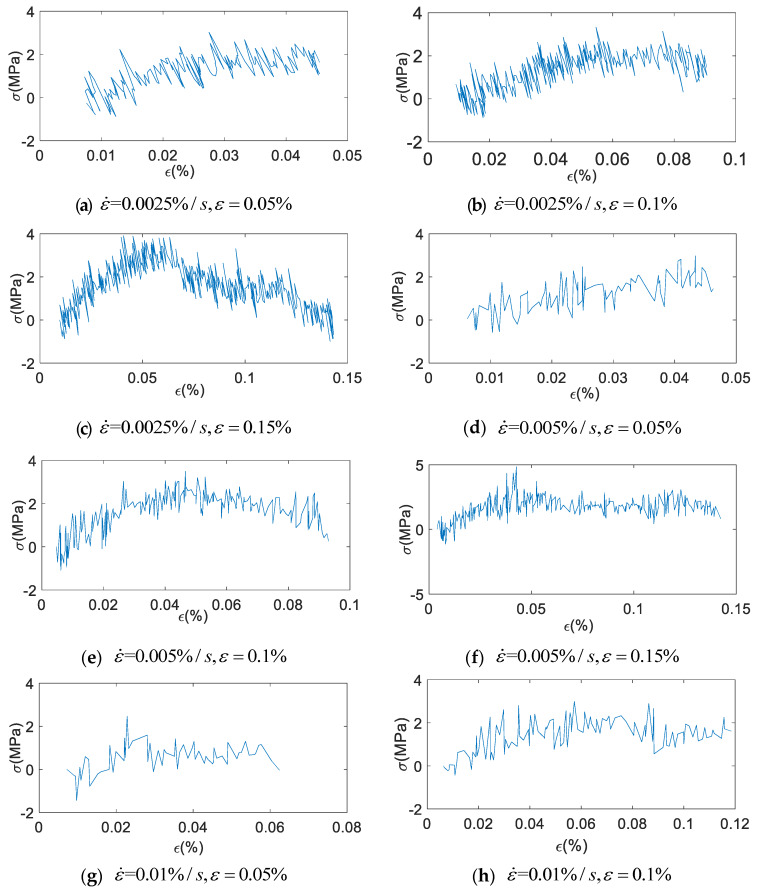

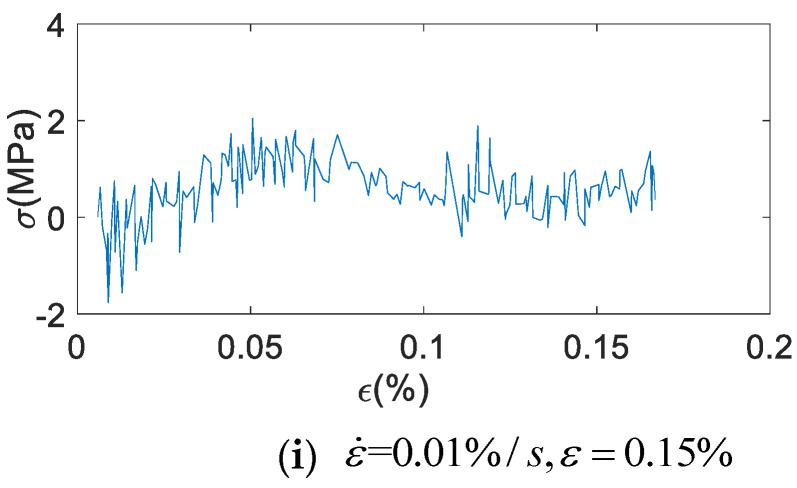
The deviation diagram of uniaxial cyclic tensile test data with the fitting curve of fractional Maxwell model in the loading stage (x-axial is strain ε (%), y-axial is stress σ (MPa), $\dot{\varepsilon }$ (%/s) is strain rate).

The fractional Maxwell model cannot simulate the hysteresis curve of damping alloys completely. Its maximum stress value of fitting curve is small. This is because of ignoring the friction between martensite and austenite or itself, that leads to an error term. So according to the difference value between the uniaxial cyclic tensile test under constant strain rate and fitting data of fractional Maxwell model, add a correction term. Figure 26 shows the error diagram of uniaxial cyclic tensile test data with the fitting curve of fractional Maxwell model in the loading stage.

As shown in Figure 26, it can be seen that the curve basically shows a sinusoidal wave peak. So the first-order sinusoidal function is used to fit, and the correction term can be obtained:

(24) Δ=asin(bε+c)

Then, changing the original fractional Maxwell constitutive Equation (6) into (25), let the stress of original fractional Maxwell model is $\sigma_{m}$:

(25) $$\sigma_{m}=\kappa D_{t}^{\alpha }\varepsilon -\frac{\kappa }{E}D_{t}^{\alpha }\sigma_{m}$$

The constitutive equation of modified fractional order Maxwell model can be obtained by adding the modified term (24):

(26) $$\sigma =\kappa D_{t}^{\alpha }\varepsilon -\frac{\kappa }{E}D_{t}^{\alpha }\sigma_{m}+\Delta$$

### 5.2. Simulation Analysis of Fitting Data

**Figure 27:**
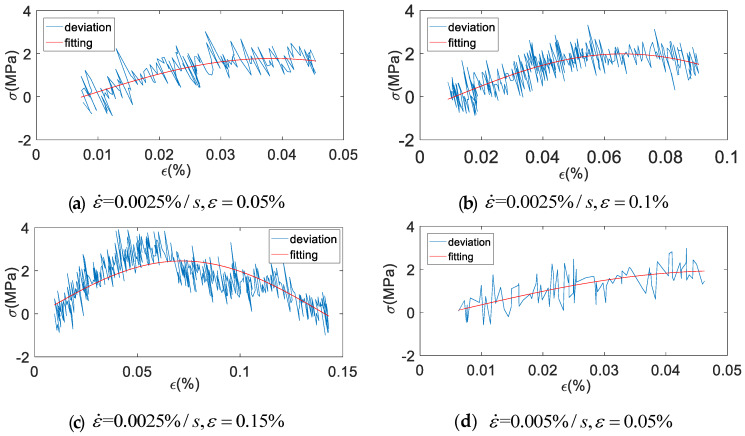

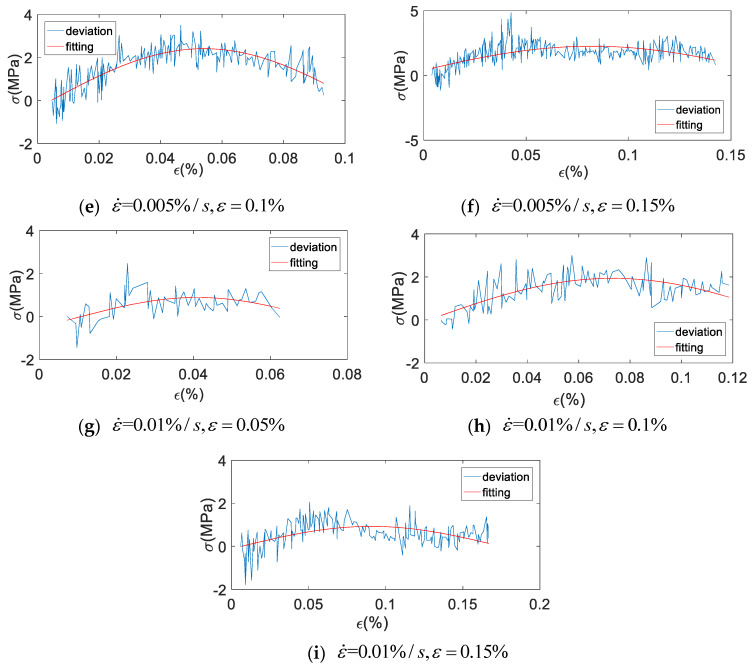
Fitting diagram of correction terms (x-axial is strain ε (%), y-axial is stress σ (MPa), $\dot{\varepsilon }$ (%/s) is strain rate, red is the fitting curve and blue is the original deviation value).

Firstly, the correction coefficients of a, b and c were fitted in MATLAB according to Equation (24), and then the fitting curve of modified fractional order Maxwell model was obtained by substituting into Equation (26). The fitting diagram of the correction term is shown in Figure 27. The fitting coefficient and evaluation index of the correction term are shown in Table 6.

**Figure 28:**
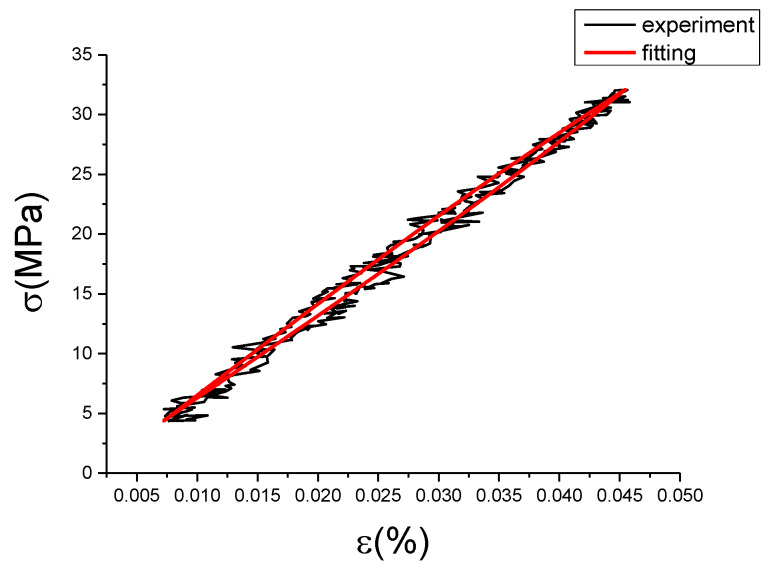
Comparison of experimental stress–strain curves and model predictions at $\dot{\varepsilon }=0.0025\%/s$ and $\varepsilon_{a}=0.05\%$. (ε(%) is strain, σ (MPa) is stress, $\dot{\varepsilon }(\%/s)$ is strain rate, $\varepsilon_{a}(\%)$ is strain amplitude, the fitting curve is red and the experiment data is black.).

**Figure 29:**
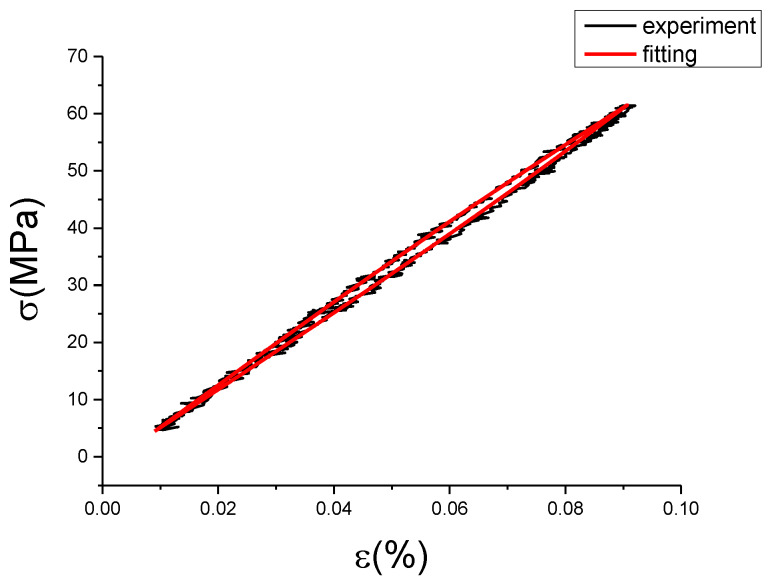
Comparison of experimental stress–strain curves and model predictions at $\dot{\varepsilon }=0.0025\%/s$ and $\varepsilon_{a}=0.1\%$. (ε(%) is strain, σ (MPa) is stress, $\dot{\varepsilon }(\%/s)$ is strain rate, $\varepsilon_{a}(\%)$ is strain amplitude, the fitting curve is red and the experiment data is black.).

**Figure 30:**
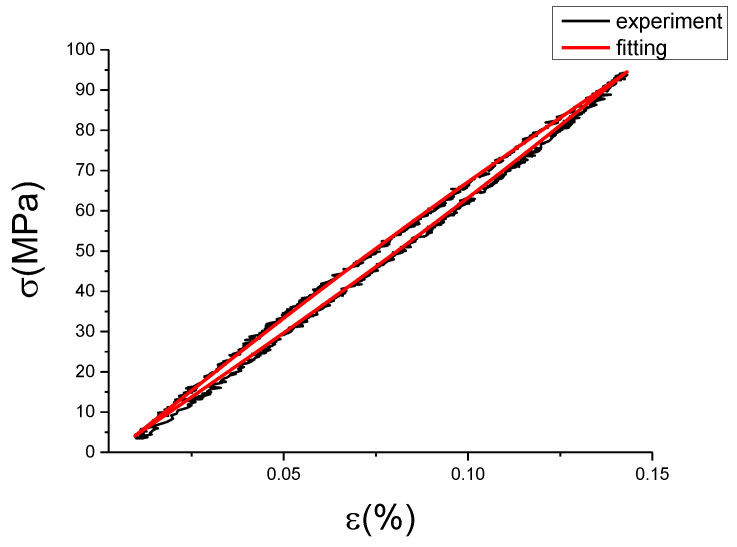
Comparison of experimental stress–strain curves and model predictions at $\dot{\varepsilon }=0.0025\%/s$ and $\varepsilon_{a}=0.15\%$. (ε(%) is strain, σ (MPa) is stress, $\dot{\varepsilon }(\%/s)$ is strain rate, $\varepsilon_{a}(\%)$ is strain amplitude, the fitting curve is red and the experiment data is black.).

**Figure 31:**
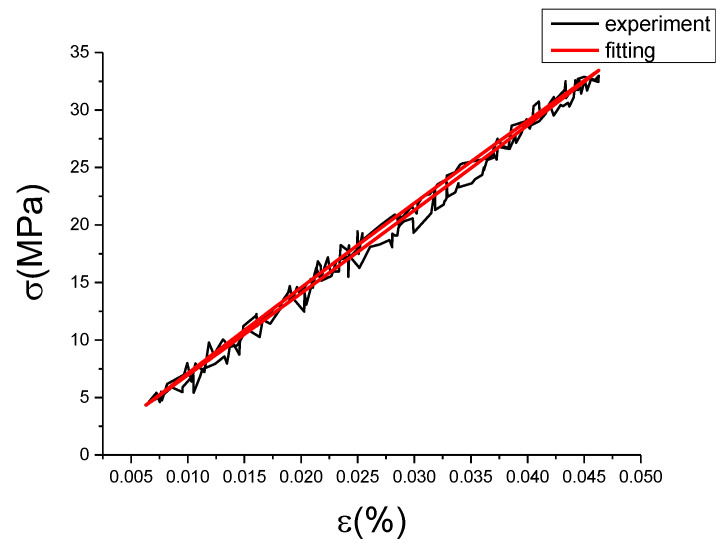
Comparison of experimental stress–strain curves and model predictions at $\dot{\varepsilon }=0.005\%/s$ and $\varepsilon_{a}=0.05\%$. (ε(%) is strain, σ (MPa) is stress, $\dot{\varepsilon }(\%/s)$ is strain rate, $\varepsilon_{a}(\%)$ is strain amplitude, the fitting curve is red and the experiment data is black.).

**Figure 32:**
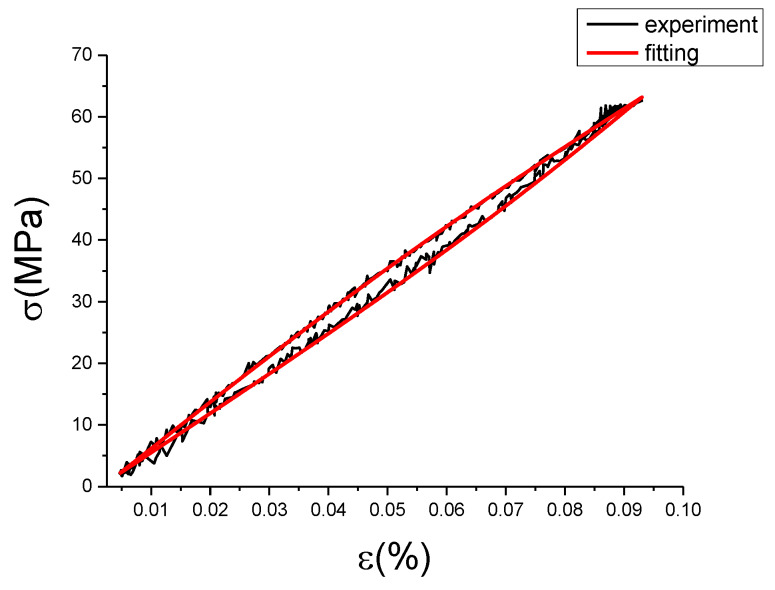
Comparison of experimental stress–strain curves and model predictions at $\dot{\varepsilon }=0.005\%/s$ and $\varepsilon_{a}=0.1\%$. (ε(%) is strain, σ (MPa) is stress, $\dot{\varepsilon }(\%/s)$ is strain rate, $\varepsilon_{a}(\%)$ is strain amplitude, the fitting curve is red and the experiment data is black.).

**Figure 33:**
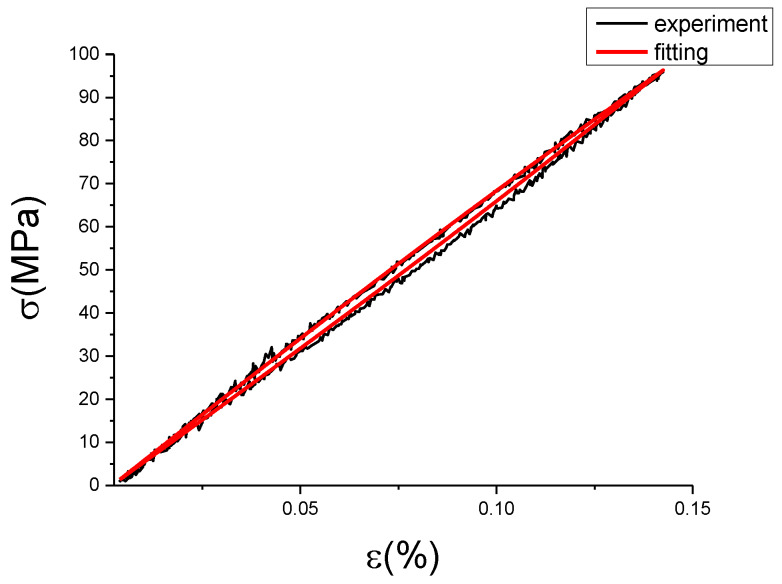
Comparison of experimental stress–strain curves and model predictions at $\dot{\varepsilon }=0.005\%/s$ and $\varepsilon_{a}=0.15\%$. (ε(%) is strain, σ (MPa) is stress, $\dot{\varepsilon }(\%/s)$ is strain rate, $\varepsilon_{a}(\%)$ is strain amplitude, the fitting curve is red and the experiment data is black.).

**Figure 34:**
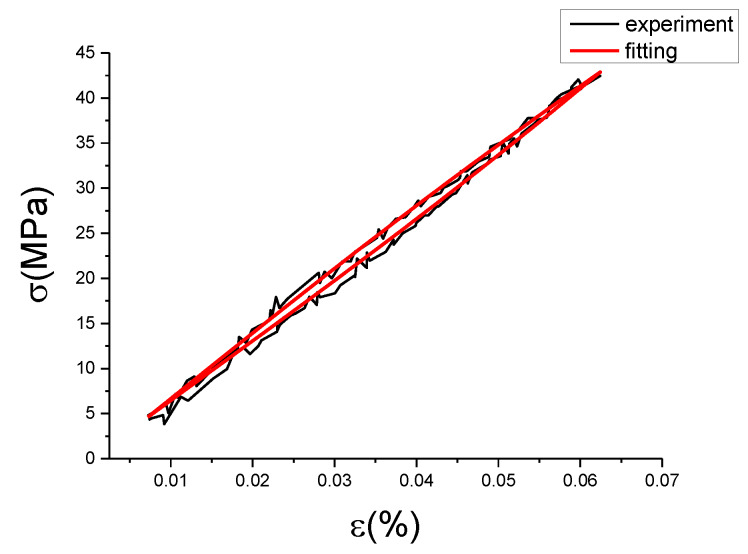
Comparison of experimental stress–strain curves and model predictions at $\dot{\varepsilon }=0.01\%/s$ and $\varepsilon_{a}=0.05\%$. (ε(%) is strain, σ (MPa) is stress, $\dot{\varepsilon }(\%/s)$ is strain rate, $\varepsilon_{a}(\%)$ is strain amplitude, the fitting curve is red and the experiment data is black.).

**Figure 35:**
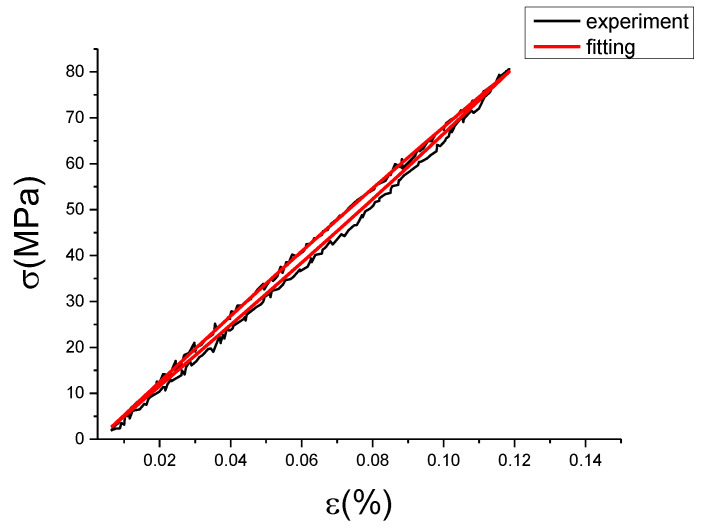
Comparison of experimental stress–strain curves and model predictions at $\dot{\varepsilon }=0.01\%/s$ and $\varepsilon_{a}=0.1\%$. (ε(%) is strain, σ (MPa) is stress, $\dot{\varepsilon }(\%/s)$ is strain rate, $\varepsilon_{a}(\%)$ is strain amplitude, the fitting curve is red and the experiment data is black.).

**Figure 36:**
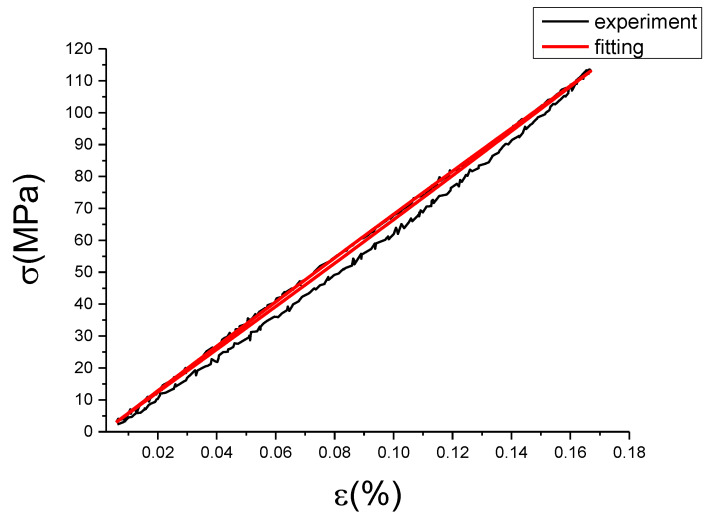
Comparison of experimental stress–strain curves and model predictions at $\dot{\varepsilon }=0.01\%/s$ and $\varepsilon_{a}=0.15\%$. (ε(%) is strain, σ (MPa) is stress, $\dot{\varepsilon }(\%/s)$ is strain rate, $\varepsilon_{a}(\%)$ is strain amplitude, the fitting curve is red and the experiment data is black.).

It can be seen from Figure 27 and Table 6 that the correction term fits the deviation value well, which basically reflects the change of the deviation value. Root of mean square (RMSE) values are all less than 0.74, and some coefficient of determination (R^2^) values are smaller, which is caused by too many points of the fitting samples. Figure 28, Figure 29, Figure 30, Figure 31, Figure 32, Figure 33, Figure 34, Figure 35 and Figure 36 are the fitting results of modified Maxwell model on experimental data.

From Figure 28, Figure 29, Figure 30, Figure 31, Figure 32, Figure 33, Figure 34, Figure 35 and Figure 36 we can see modified fractional Maxwell model fitting is better with the test data, and can clearly show the nonlinear constitutive relation of damping alloy. The hysteresis area is obvious, and the fitting curve is smoother than test data. This is convenient to be used in analysis. The loading and unloading curve of modified fractional Maxwell model fitting are symmetry about the center line. However, the stress-strain fitting curve at the strain rate of 0.01%/s and the strain amplitude of 0.15% has a small hysteresis area, and the loading section is well fitted, but the unloading section has a large deviation, which is due to the error of experimental data collection. Table 7 is the evaluation index of modified fractional Maxwell model corresponding to each group of data.

**Figure 37:**
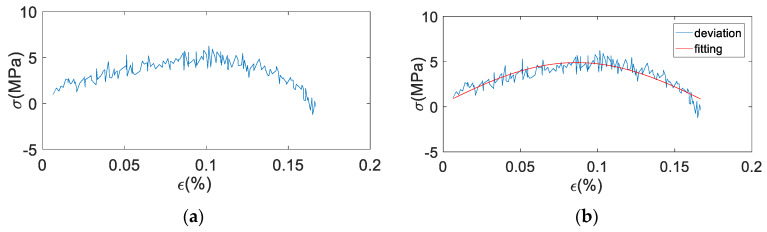
The unloading deviation curve and the unloading correction term fitting curve. (**a**) the unloading deviation curve; (**b**) unloading correction term fitting curve.

It can be seen from Table 7 that the modified fractional Maxwell model has a good fitting effect on the experimental data, and the determination coefficients can reach above 0.99, but the mean square error and variance of group 9 are large. Since its fitting deviation is mainly in the unloading section, try to set the unloading period of experiment data as object of correction fitting program. First, we can use the coefficient and the model of the fractional Maxwell to calculate the fitting curve of unloading stage. Then we can use it and the unloading stage data of the test to fit the modified term. Finally, the loading stage was calculated according to the modified fractional Maxwell model. The unloading deviation curve and the unloading correction term fitting curve were shown in Figure 37a,b.

**Figure 38:**
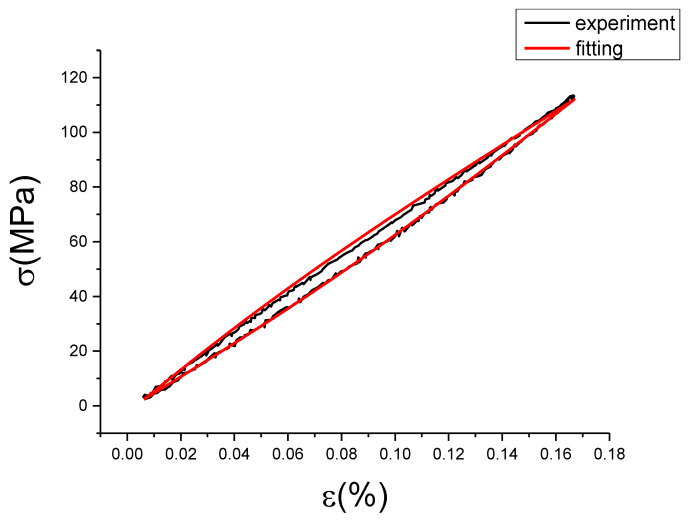
The modified fractional Maxwell model curve fitted according to the unloading section compared with the experiment data at $\dot{\varepsilon }=0.01\%/s$ and $\varepsilon_{a}=0.15\%$. (ε(%) is strain, σ (MPa) is stress, $\dot{\varepsilon }(\%/s)$ is strain rate, $\varepsilon_{a}(\%)$ is strain amplitude, the fitting curve is red and the experiment data is black.).

Coefficient and evaluation index of the unloaded correction term: a = 4.886, b = 17.34, c = 0.07346, SSE (variance) = 85.89, R^2^ (determination coefficient) = 0.7703, RMS (mean square root) = 0.6966. The modified fractional Maxwell model curve fitted according to the unloading section compared with the experiment data is shown in Figure 38.

The fitting curve in Figure 38 compared with the test data concluded that MSE = 1.363, SSE = 490.673, R^2^ = 0.9990, also it can be seen from the picture that effect is superior to the original loading correction fractional fitting. This reason should be caused by the error generated in test collection, suggesting non-linear of unloading stage is better. This has confirmed that modified fractional Maxwell for this strain rate and strain amplitude is feasible.

In order to show the applicability of modified fractional Maxwell model, a comparison of fitting performance between the modified fractional Maxwell model and other models was be done. Choosing the stress-strain curve of experiment at 0.005%/s and 0.1% as the fitting standard.

According to Boltzmann superposition principle [34], the stress of material can be express by Equation (27).

(27) $$\begin{array}{ll} \sigma (t) & =\varepsilon_{0}G(t)+\int_{0}^{t}G(t-s)\dot{\varepsilon }ds\\ & =G(t)*d\varepsilon (t) \end{array}$$

where $\varepsilon_{0}$ is the Initial stain value, G(t) is the relaxation modulus, $\dot{\varepsilon }$ is the strain rate, “∗” is the symbol of Stieltjes convolution [34].

**Figure 39:**
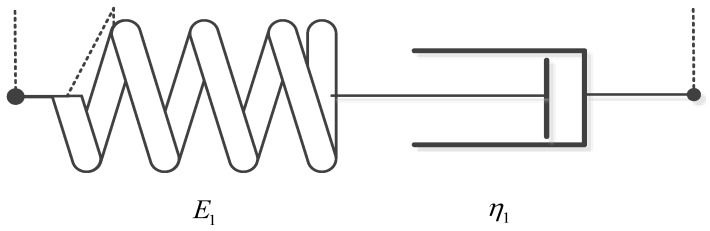
The schematic of classic Maxwell model.

For the classical Maxwell model (Maxwell two-parameter model), as shown in Figure 39.

(28) $$G(t)=E_{1}e^{-\frac{t}{\tau_{1}}}$$

where *E*_1_ is the elastic modulus, $\tau_{1}=\frac{\eta_{1}}{E_{1}}$ is the relaxation time.

Since the $\dot{\varepsilon }$ is constant, take Equation (28) into (27) can get:

(29) $$\begin{array}{ll} \sigma (t) & =\varepsilon_{0}E_{1}e^{-\frac{t}{\tau_{1}}}+\dot{\varepsilon }\int_{0}^{t}E_{1}e^{-\frac{t-s}{\tau_{1}}}ds\\ & =\varepsilon_{0}E_{1}e^{-\frac{t}{\tau_{1}}}+E_{1}\dot{\varepsilon }\tau_{1}-E_{1}\dot{\varepsilon }\tau_{1}e^{-\frac{t}{\tau_{1}}} \end{array}$$

**Figure 40:**
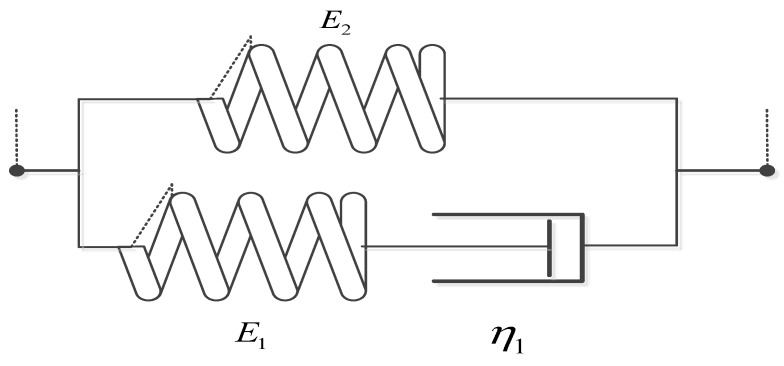
The schematic of Maxwell 3 parameters model.

For the Maxwell three-parameter model, as shown in Figure 40.

(30) $$G(t)=E_{1}e^{-\frac{t}{\tau_{1}}}+E_{2}$$

where E_1_ and E_2_ are the elastic modulus, $\tau_{1}=\frac{\eta_{1}}{E_{1}}$ is the relaxation time.

Since the $\dot{\varepsilon }$ is constant, take Equation (30) into (27) can get:

(31) $$\begin{array}{ll} \sigma (t) & =\varepsilon_{0}G(t)+\int_{0}^{t}G(t-s)\dot{\varepsilon }ds\\ & =\varepsilon_{0}\left(E_{1}e^{-\frac{t}{\tau_{1}}}+E_{2}\right)+\int_{0}^{t}\left(E_{1}e^{-\frac{t-s}{\tau_{1}}}+E_{2}\right)\dot{\varepsilon }ds\\ & =\varepsilon_{0}\left(E_{1}e^{-\frac{t}{\tau_{1}}}+E_{2}\right)+E_{2}\dot{\varepsilon }t+E_{1}\dot{\varepsilon }\tau_{1}-E_{1}\dot{\varepsilon }\tau_{1}e^{-\frac{t}{\tau_{1}}}\\ & =\left(E_{1}\varepsilon_{0}-E_{1}\dot{\varepsilon }\tau_{1}\right)e^{-\frac{t}{\tau_{1}}}+E_{2}\varepsilon_{0}+E_{2}\dot{\varepsilon }t+E_{1}\dot{\varepsilon }\tau_{1} \end{array}$$

**Figure 41:**
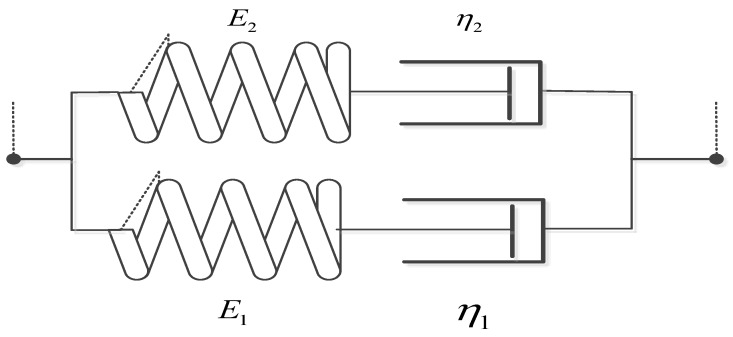
The schematic of Maxwell four-parameter model.

For the Maxwell four-parameter model, as shown in Figure 41.

(32) $$G(t)=E_{1}e^{-\frac{t}{\tau_{1}}}+E_{2}e^{-\frac{t}{\tau_{2}}}$$

where E_1_ and E_2_ are the elastic modulus, $\tau_{1}=\frac{\eta_{1}}{E_{1}}$ and $\tau_{2}=\frac{\eta_{2}}{E_{2}}$ are the relaxation time.

Since the $\dot{\varepsilon }$ is constant, take Equation (32) into (27) can get:

(33) $$\begin{array}{ll} \sigma (t) & =\varepsilon_{0}G(t)+\int_{0}^{t}G(t-s)\dot{\varepsilon }ds\\ & =\varepsilon_{0}\left(E_{1}e^{-\frac{t}{\tau_{1}}}+E_{2}e^{-\frac{t}{\tau_{2}}}\right)+\int_{0}^{t}\left(E_{1}e^{-\frac{t-s}{\tau_{1}}}+E_{2}e^{-\frac{t-s}{\tau_{2}}}\right)\dot{\varepsilon }ds\\ & =\varepsilon_{0}\left(E_{1}e^{-\frac{t}{\tau_{1}}}+E_{2}e^{-\frac{t}{\tau_{2}}}\right)+E_{1}\dot{\varepsilon }\tau_{1}-E_{1}\dot{\varepsilon }\tau_{1}e^{-\frac{t}{\tau_{1}}}+E_{2}\dot{\varepsilon }\tau_{2}-E_{2}\dot{\varepsilon }\tau_{2}e^{-\frac{t}{\tau_{2}}}\\ & =\left(E_{1}\varepsilon_{0}-E_{1}\dot{\varepsilon }\tau_{1}\right)e^{-\frac{t}{\tau_{1}}}+\left(E_{2}\varepsilon_{0}-E_{2}\dot{\varepsilon }\tau_{2}\right)e^{-\frac{t}{\tau_{2}}}+E_{1}\dot{\varepsilon }\tau_{1}+E_{2}\dot{\varepsilon }\tau_{2} \end{array}$$

**Figure 42:**
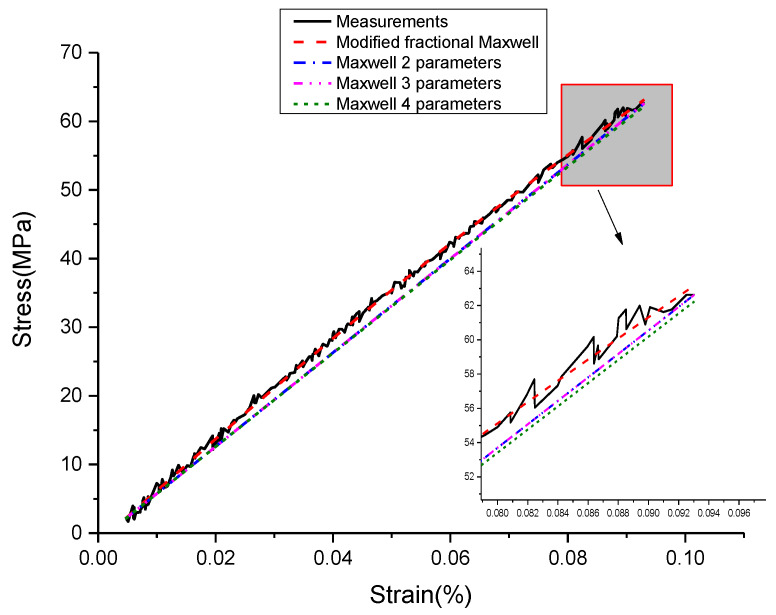
The optimal Maxwell models fit to the loading experimental data at $\dot{\varepsilon }=0.005\%/s$ and $\varepsilon_{a}=0.1\%$. ($\dot{\varepsilon }(\%/s)$ is strain rate, $\varepsilon_{a}(\%)$ is strain amplitude.).

Next, least-squares approximations are used, and two-, three- and four-parameter optimal models were determined. The parameters and evaluation index of Maxwell models are given in Table 8. Figure 42 presents experiment data and the optimal Maxwell models.

It is easy to observe, that the classical Maxwell model, Maxwell three-parameter model or Maxwell four-parameter model are inappropriate for description of this nonlinearity of elastic loading process. A better fit to experimental data can be obtained, if the modified fractional Maxwell model is used. In addition to, because the E1 of Maxwell three-parameter model is too small that is almost equal to zero, the fitting curve of Maxwell two-parameter model is close to Maxwell three-parameter model shown in the Figure 39 and Table 8. All this has confirmed that the modified fractional Maxwell can well explain the nonlinear constitutive relation of damping alloy.

Since the parameters of the modified Maxwell model need to be determined according to the different conditions of each load, the application of this model is limited. In order to improve the usability of the model, the relationship between model parameters and loading conditions is analyzed. By using the method, which calculated the average value of the parameters at different strain rates but same strain amplitude, the influence of strain rate was eliminated, and the variation rule between strain amplitude and each parameter was obtained. Then, a strain-related formula is proposed for each parameter, so that the parameters of the modified Maxwell model can be determined and the model can maintain at a high precision under the condition that the particular experiment cannot be carried out.

However, the first step is to assess the impact of replacing the original parameter with the mean value of parameter. According to the values of parameters under different strain rates and strain amplitudes, the parameters under the same strain amplitudes but different strain rates were averaged, as shown in Table 9. This way can minimize the impact of strain rates on model parameters, and get the functions of model parameters, which are only related to strain.

For evaluating the impact of the average value of each parameter on the accuracy of model fitting, the average value (Table 10) was used to replace the original model parameters, and substituted into MATLAB. The fitting effect is expressed in figures of error, as shown in Table 11.

**Figure 43:**
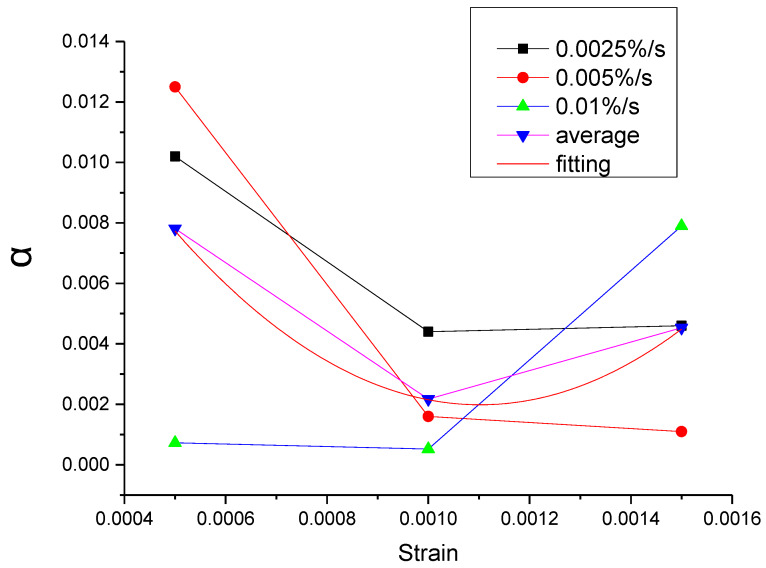
The value of α at different conditions and the fitting of the mean value of α.

**Figure 44:**
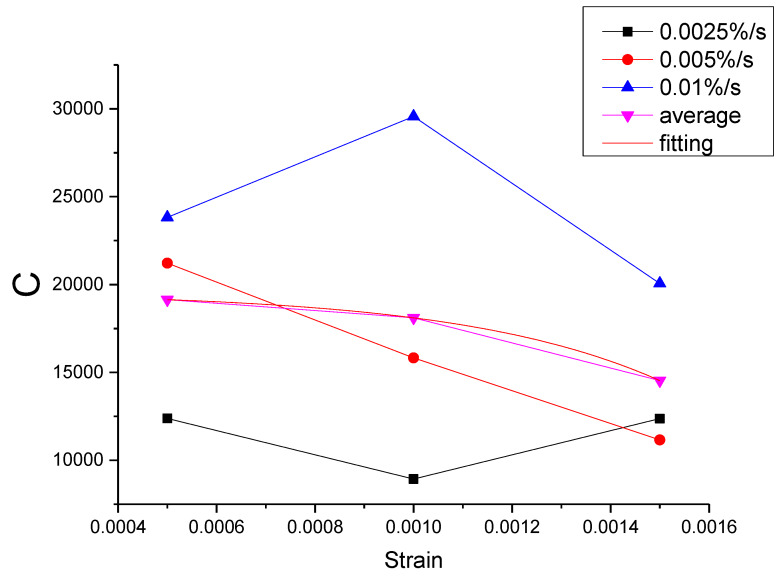
The value of C at different conditions and the fitting of the mean value of C.

**Figure 45:**
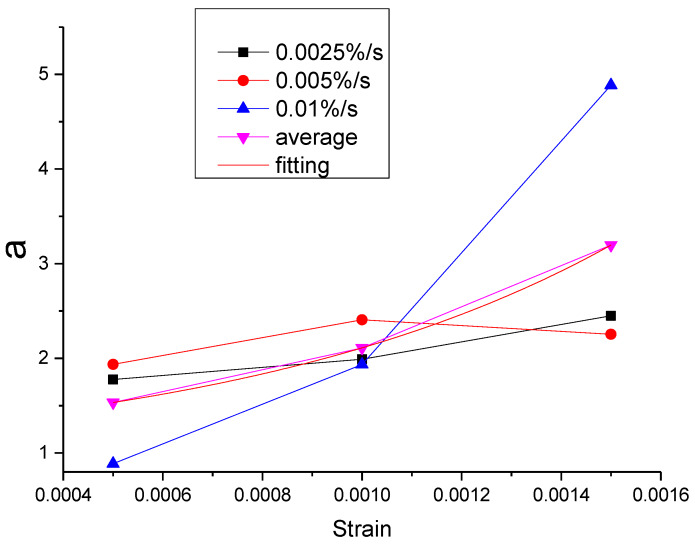
The value of a at different conditions and the fitting of the mean value of a.

**Figure 46:**
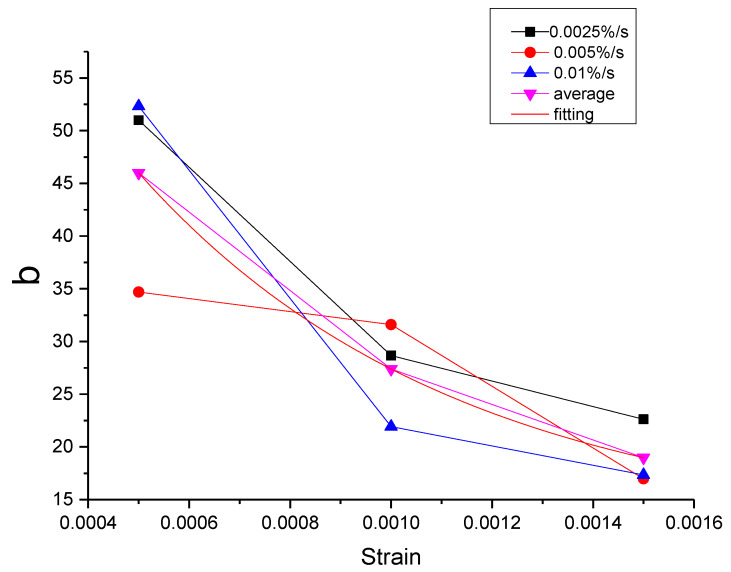
The value of b at different conditions and the fitting of the mean value of b.

**Figure 47:**
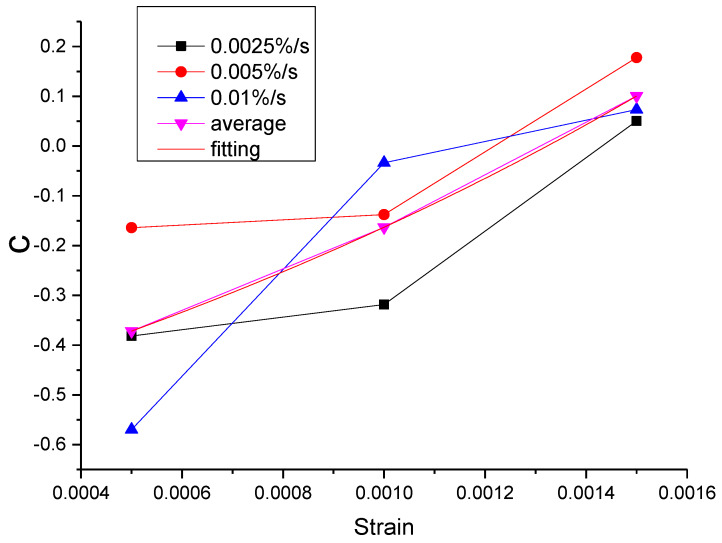
The value of c at different conditions and the fitting of the mean value of c.

As can be seen from Table 11, the errors of the mean parameters fitting relative to the test and the original parameters fitting are all within the acceptable range. The R^2^ values between mean parameters fitting curve and test are all above 0.9954, while the R^2^ values between mean parameters fitting curve and the original parameters fitting curve are all above 0.9996. Therefore, the influence of the strain rate can be ignored and the mean parameters can be used to replace the original parameters. Then, the function of each parameter can be proposed according to the relationship between the mean parameter and the strain, as shown in Figure 43, Figure 44, Figure 45, Figure 46 and Figure 47.

The fitting function of the relation between each parameter and strain is shown in Table 12.

It can be seen from Figure 43, Figure 44, Figure 45, Figure 46 and Figure 47 that the equations in Table 12 all have a good fitting result for the change of each parameter with strain. What is interesting is that all the functions of parameters can be expressed as exponential functions, except for α. Therefore, the functions in Table 12 can be used to calculate the values of model parameters under other loading conditions within the elastic range, instead of the constant strain rate tensile test, which extends the practicability of the modified Maxwell model.

## 6. Conclusions

In view of the existing study about nonlinear constitutive relation of Mn-Cu damping alloy being less, and that it should not be treated as linear elastic material, this study chose M2052 damping alloy as the research object, through uniaxial cyclic tensile test under constant strain rate to analysis its nonlinear constitutive relation and hysteretic characteristics. By considering the damping alloy as a special viscoelastic material and based on the fractional Maxwell model which describes nonlinear viscoelasticity, a modified fractional Maxwell model suitable for M2052 damping alloy was proposed. Through the numerical simulation, we can get the following conclusions:

1. Through uniaxial cyclic tensile test with constant strain rate, it is concluded that Mn-Cu damping alloy can be considered as a viscoelastic material. In the elastic strain range, as the strain amplitude increases, the slope of the stress-strain curve decreases and the hysteresis loop area and the damping capacity increases with the same strain rate. Under the same strain amplitude, the slope and hysteresis area of the stress-strain curve change with the change of the strain rate, this still needs further study.

2. The Fractional Maxwell model only contains two unknown coefficients and does not have to get analytic solutions. It can combine with uniaxial cyclic tensile test under constant strain rate and relaxation test to determine its parameters. Since the max-stress of loading stage is biggish, the fractional coefficient should be decreased and the quasi-state coefficient should be increased to achieve the minimum mean square error. This leads to weak nonlinear fitting curve, which does not match the nonlinear characteristics of damping alloy. However, the fitting curve of fractional Maxwell model is basically in the middle of the loading and unloading stress-strain curve, which can be used in some engineering application or situations where the accuracy is not high.

3. The error of fractional Maxwell model is due to ignoring the friction of new martensite with austenite and original martensite. Combining with the deviation curve, a five-variable modified fractional Maxwell phenomenological model is proposed. This model can well simulate the nonlinear characteristics and the hysteresis curve of damping alloys. Determine coefficients all above 0.99, and the deviation is small. Data fluctuation and measurement error exist in the test curve. However, the fitting curves do not have these problems and can fully capture the stress-strain variation trend of the damping alloy. Through compare with other Maxwell models, it also shows a better fitting performance. In addition, a strain-related formula is proposed for each parameter, so that the parameters of the modified Maxwell model can be determined without the particular experiment. Therefore, the modified fractional Maxwell model can be used as the constructive equation of Mn-Cu damping alloys and reference for the further analysis.

## Figures and Tables

**Table 1:** Natural properties of M2052 damping alloy.

Name	Tensile Strength	Young’s Modulus	Poisson’s Ratio	Yield Strength	Density
**Value**	540 MPa	68.5 GPa	0.338	205 MPa	7.25 g/cm^3^

**Table 2:** Hysteresis area under different strain rates and strain amplitudes (unit: 10 kJ/m^3^).

Strain Amplitudes		0.05%	0.1%	0.15%
**Strain Rates**	**0.0025%/s**	0.041872	0.147943	0.503593
	**0.005%/s**	0.036426	0.180372	0.3791682
	**0.01%/s**	0.071483	0.319285	0.653956

**Table 3:** The slope of curve under different strain rates and strain amplitudes.

Strain Amplitudes		0.05%	0.1%	0.15%
**Strain Rates**	**0.0025%/s**	730.61979	687.76791	669.23966
	**0.005%/s**	724.0259	694.45375	680.06215
	**0.01%/s**	703.77106	689.28956	681.57759

**Table 4:** The strain amplitude error between the set value and the measured value.

Number	$\dot{\varepsilon }(\%/s)$	$\varepsilon_{\mathrm{set}}(\%)$	$\varepsilon_{measured}\left(\%\right)$	Error (%)	The Relative Error (%)
**1**	0.0025	0.05%	0.04748	0.00437	0.0504
**2**		0.1%	0.09132	0.00797	0.0868
**3**		0.15%	0.14239	0.00681	0.05073
**4**	0.005	0.05%	0.04332	0.00372	0.1336
**5**		0.1%	0.09123	0.00696	0.0877
**6**		0.15%	0.14132	0.00779	0.05787
**7**	0.01	0.05%	0.05668	−0.01243	−0.1336
**8**		0.1%	0.11754	−0.01848	−0.1754
**9**		0.15%	0.1558	−0.01686	−0.03867

**Table 5:** Fractional Maxwell model fitting coefficient.

Number	$\dot{\varepsilon }(\%/s)$	ε(%)	Generations	Fval	α	C
**1**	0.0025	0.05	18,191	1.9510	0.0102	12,380.56
**2**		0.1	11,591	1.123	0.0044	8929
**3**		0.15	19,989	3.8929	0.0046	12,374
**4**	0.005	0.05	28,735	2.0648	0.0125	21,216.625
**5**		0.1	20,263	3.98	0.0016	15,824.938
**6**		0.15	10,449	3.92	0.0011	11,160.78
**7**	0.01	0.05	34,455	0.7885	7.2797 × 10^−4^	23,822
**8**		0.1	44,922	2.6657	5.1945× 10^−4^	29,563
**9**		0.15	27,016	0.7995	0.0079	20,059.3

**Table 6:** Fitting coefficient and evaluation index of the modified item.

Number	$\dot{\varepsilon }(\%/s)$	ε(%)	a	b	c	SSE (Variance)	R^2^ (Determinate Coefficient)	RMSE (Mean Square Root)
**1**	0.0025	0.05%	1.777	50.99	−0.3816	0.0037	0.9999	0.0046
**2**		0.1%	1.99	28.66	−0.3183	1.47	0.9917	0.0609
**3**		0.15%	2.45	22.61	0.0504	258.2	0.5804	0.579
**4**	0.005	0.05%	1.937	34.69	−0.164	30.99	0.4756	0.5836
**5**		0.1%	2.408	31.6	−0.1376	60.24	0.6412	0.5488
**6**		0.15%	2.255	16.97	0.1778	162.3	0.3146	0.7405
**7**	0.01	0.05%	0.8868	52.32	−0.5697	17.88	0.2591	0.5504
**8**		0.1%	1.935	21.92	−0.0335	34.78	0.4599	0.5407
**9**		0.15%	0.9247	18.7	−0.1211	56.27	0.2077	0.5639

**Table 7:** The evaluation index of modified fractional Maxwell model.

Number	$\dot{\varepsilon }(\%/s)$	ε(%)	SSE (Variance)	RMSE (Mean Square Root)	R^2^ (Determinate Coefficient)
**1**	0.0025	0.05	181.0160	0.4897	0.9933
**2**		0.1	324.4874	0.3986	0.9989
**3**		0.15	986.723	0.8313	0.9991
**4**	0.005	0.05	101.1080	0.5266	0.9938
**5**		0.1	286.8182	0.6895	0.9983
**6**		0.15%	643.7925	1.0537	0.999
**7**	0.01	0.05	57.6222	0.4237	0.9972
**8**		0.1	277.4389	1.0837	0.9986
**9**		0.15	1843.8	5.1216	0.9975

**Table 8:** Parameters and evaluation index of Maxwell models.

Number	Name	E_1_ (GPa)	E_2_ (GPa)	$\tau_{1}(s)$	$\tau_{2}(s)$	SSE (Variance)	R^2^ (Determinate Coefficient)	RMSE (Mean Square Root)
**1**	Two-parameter model	68.5	-	3.993 × 10^5^	-	1.926 × 10^8^	0.9969	976.5
**2**	Three-parameter model	2.22 × 10^−14^	68.5	75.06	-	1.925 × 10^8^	0.9969	976.1
**3**	Four-parameter model	1.243	67.257	1205	1503	2.235 × 10^8^	0.9964	1057

**Table 9:** The average values of model parameters under the same strain amplitudes but different strain rates.

Number	Strain Amplitude (%)	Strain Rate (%/s)	α	C	a	b	c
**1**	0.05%	0.0025	0.0102	12,380.56	1.777	50.99	−0.3816
**2**		0.005	0.0125	21,216.625	1.937	34.69	−0.164
**3**		0.01	7.28 × 10^−4^	23,822	0.8868	52.32	−0.5697
**4**	**Average**		0.007809	19,139.72833	1.5336	46	−0.371767
**5**	0.10%	0.0025	0.0044	8929	1.99	28.66	−0.3183
**6**		0.005	0.0016	15,824.938	2.408	31.6	−0.1376
**7**		0.01	5.19 × 10^−4^	29,563	1.935	21.92	−0.0335
**8**	**Average**		0.002173	18,105.65	2.111	27.39333	−0.16313
**9**	0.15%	0.0025	0.0046	12,374	2.45	22.61	0.0504
**10**		0.005	0.0011	11,160.78	2.255	16.97	0.1778
**11**		0.01	0.0079	20,059.3	4.886	17.34	0.07346
**12**	**Average**		0.004533	14,531.36	3.197	18.97333	0.100553

**Table 10:** Model parameters under different loading conditions after averaging.

Number	Strain Rate (%/s)	Strain Amplitude (%)	α	C	a	b	c
**1**	0.0025	0.05	0.007809	19,139.72833	1.5336	46	−0.371767
**2**		0.1	0.002173	18,105.65	2.111	27.39333	−0.16313
**3**		0.15	0.004533	14,531.36	3.197	18.97333	0.100553
**4**	0.005	0.05	0.007809	19,139.72833	1.5336	46	−0.371767
**5**		0.1	0.002173	18,105.65	2.111	27.39333	−0.16313
**6**		0.15	0.004533	14,531.36	3.197	18.97333	0.100553
**7**	0.01	0.05	0.007809	19,139.72833	1.5336	46	−0.371767
**8**		0.1	0.002173	18,105.65	2.111	27.39333	−0.16313
**9**		0.15	0.004533	14,531.36	3.197	18.97333	0.100553

**Table 11:** The error of mean parameters fitting curve with experimental curve and original model parameter fitting curve.

Number	$\dot{\varepsilon }(\%/s)$	ε(%)	MSE	SSE	R^2^	MSE	SSE	R^2^
			Compared with Experimental Curve			Compared with Original Model Parameter Fitting Curve		
**1**	0.0025	0.05%	0.3401	59.5182	0.9955	0.0493	8.6320	0.9999
**2**		0.1%	0.3633	145.3041	0.9991	0.1087	43.4667	0.9999
**3**		0.15%	1.2377	736.4596	0.9994	0.7022	417.7823	0.9999
**4**	0.005	0.05%	0.3900	36.6571	0.9954	0.0600	5.6439	0.9999
**5**		0.1%	0.4237	86.0018	0.9988	0.1286	26.1143	0.9998
**6**		0.15%	1.0439	312.1178	0.9992	0.5027	150.2989	0.9999
**7**	0.01	0.05%	0.5603	34.7357	0.9976	0.2715	16.8323	0.9999
**8**		0.1%	0.4168	50.8456	0.9993	0.1352	16.4890	0.9999
**9**		0.15%	2.7624	497.2249	0.9993	2.4484	440.7147	0.9996

**Table 12:** The fitting function of each parameter.

Number	Name	Function
**1**	α	$\alpha =0.02123-34.9088\varepsilon +15832.7862\times \varepsilon^{2}$
**2**	C	$C=-121.78885\times e^{\left(\frac{\varepsilon }{4.03144\times 10^{-4}}\right)}+19560.68917$
**3**	a	$a=0.34852\times e^{\left(\frac{\varepsilon }{7.91488\times 10^{-4}}\right)}+0.87809$
**4**	b	$b=75.10374\times e^{\left(-\frac{\varepsilon }{6.30589\times 10^{-4}}\right)}+12.01361$
**5**	c	$c=0.62557\times e^{\left(\frac{\varepsilon }{0.00214}\right)}-1.16242$

## Supplementary Materials

The following are available online at https://www.mdpi.com/1996-1944/13/9/2020/s1. Figure S1: Hysteresis measured curves with different strain amplitudes at strain rate of 0.0025%/s (σ is stress, ε is strain, $\varepsilon_{a}$ is strain amplitude); Figure S2: Hysteresis measured curves with different strain amplitudes at strain rate of 0.005%/s (σ is stress, ε is strain, $\varepsilon_{a}$ is strain amplitude); Figure S3: Hysteresis measured curves with different strain amplitudes at strain rate of 0.01%/s (σ is stress, ε is strain, $\varepsilon_{a}$ is strain amplitude); Table S1: Hysteresis area of measured under different strain rates and strain amplitudes (unit: 10 kJ/m^3^); Table S2: The slope of measured curve under different strain rates and strain amplitudes.
