# Supplementary material for: A Modified Fractional Maxwell Numerical Model for Constitutive Equation of Mn-Cu Damping Alloy
Source: Materials (Basel). 2020 Apr 26;13(9):2020. doi: 10.3390/ma13092020 (PMC7254341; doi:10.3390/ma13092020)
Supplement: Supplementary file 1 [file materials-13-02020-s001.pdf]

materials

Supplementary Materials

# A Modified Fractional Maxwell Numerical Model for Constitutive Equation of Mn-Cu Damping Alloy

**Baoquan Mao, Rui Zhu *, Zhiqian Wang, Yuying Yang, Xiaoping Han and Qijin Zhao**

Department of Weapons and Control Engineering, Army Academy of Armored Forces, Beijing 100072, China; mbq_1965@163.com (B.M.); wangzhiqian_1990@163.com (Z.W.); lingyuxuan2012@163.com (Y.Y.); 15811060576@163.com (X.H.); zqj563954008@163.com (Q.Z.)

* Correspondence: zhuliang@cip.com.cn

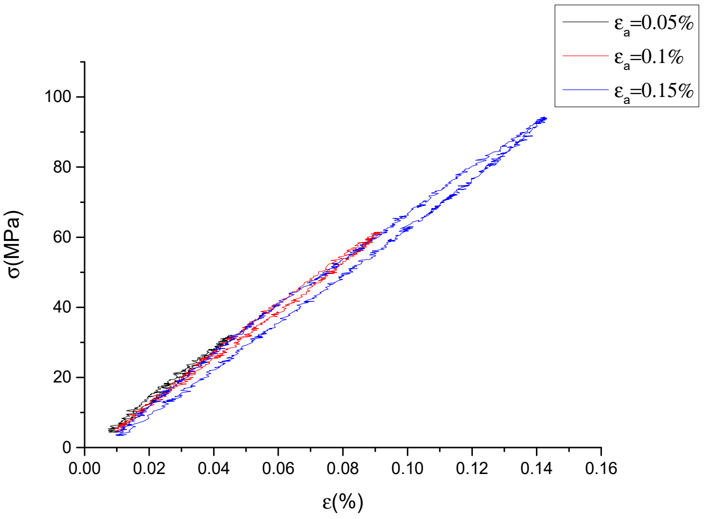


**Figure S1.** Hysteresis measured curves with different strain amplitudes at strain rate of 0.0025%/s ( $\sigma$ is stress, $\varepsilon$ is strain, $\varepsilon_a$ is strain amplitude).

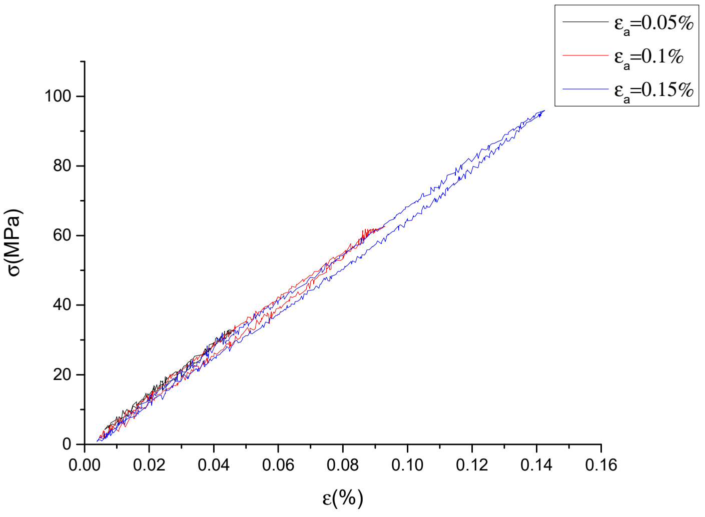


**Figure S2.** Hysteresis measured curves with different strain amplitudes at strain rate of 0.005%/s ( $\sigma$ is stress, $\varepsilon$ is strain, $\varepsilon_a$ is strain amplitude).

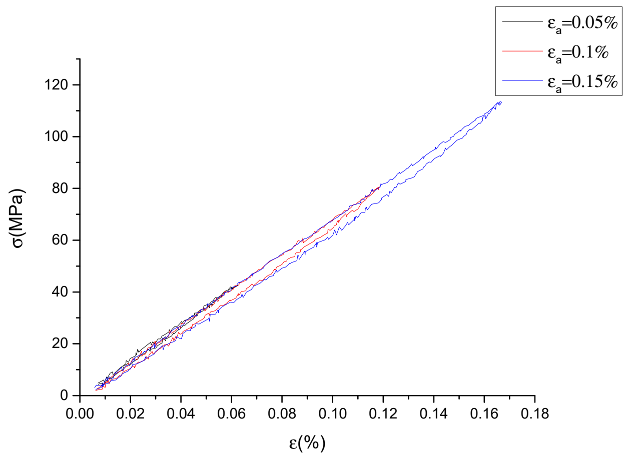


**Figure S3.** Hysteresis measured curves with different strain amplitudes at strain rate of 0.01%/s ($\sigma$ is stress, $\varepsilon$ is strain, $\varepsilon_a$ is strain amplitude).

**Table S1.** Hysteresis area of measured under different strain rates and strain amplitudes (unit: 10 kJ/m$^3$).

| **Strain Amplitudes** / **Strain Rates** | **0.05%** | **0.1%** | **0.15%** |
| --- | --- | --- | --- |
| 0.0025%/s | 0.0451 | 0.1528 | 0.5102 |
| 0.005%/s | 0.0378 | 0.1822 | 0.3810 |
| 0.01%/s | 0.0762 | 0.3161 | 0.6567 |

**Table S2.** The slope of measured curve under different strain rates and strain amplitudes.

| **Strain Amplitudes** / **Strain Rates** | **0.05%** | **0.1%** | **0.15%** |
| --- | --- | --- | --- |
| 0.0025%/s | 727.6510 | 687.1823 | 682.3001 |
| 0.005%/s | 724.5884 | 694.4415 | 680.1142 |
| 0.01%/s | 704.7360 | 689.3458 | 682.1142 |
